# Supplementary material for: Osteocytes directly regulate osteolysis via MYD88 signaling in bacterial bone infection
Source: Nat Commun. 2022 Nov 4;13:6648. doi: 10.1038/s41467-022-34352-z (PMC9636212; doi:10.1038/s41467-022-34352-z)
Supplement: Supplementary file 1 — Supplementary Information [file 41467_2022_34352_MOESM1_ESM.pdf]

Supplementary Information

Osteocytes directly regulate osteolysis via MYD88 signaling in bacterial bone infection

Tetsuya Yoshimoto[1, 2], Mizuho Kittaka[1, 2], Andrew Anh Phuong Doan[1, 2], Rina Urata[1, 2], Matthew Prideaux [2, 3], Roxana E. Rojas[4], Clifford V. Harding[5], W. Henry Boom[5, 6, 7], Lynda F. Bonewald[2, 3], Edward M. Greenfield[2, 3, 8], Yasuyoshi Ueki[1, 2 *]

[1]Department of Biomedical Sciences and Comprehensive Care, Indiana University School of Dentistry, Indianapolis, Indiana 46202-5126, USA

[2]Indiana Center for Musculoskeletal Health, Indiana University School of Medicine, Indianapolis, Indiana 46202-5126, USA

[3]Department of Anatomy, Cell Biology, and Physiology, Indiana University School of Medicine, Indianapolis, Indiana 46202-5126, USA

[4]Janssen Biopharma Inc, Brisbane, California 94005-1809, USA

[5]Department of Pathology, Case Western Reserve University & University Hospitals Cleveland Medical Center, Cleveland, Ohio 44106-4960, USA

[6]Department of Medicine, Case Western Reserve University & University Hospitals Cleveland Medical Center, Cleveland, Ohio, 44106-4960, USA

[7]Department of Molecular Biology and Microbiology, Case Western Reserve University & University Hospitals Cleveland Medical Center, Cleveland, Ohio, 44106-4960, USA

[8]Department of Orthopaedic Surgery, Indiana University School of Medicine, Indianapolis, Indiana 46202-5126, USA

[*] Corresponding author and lead contact

Supplementary Figure 1

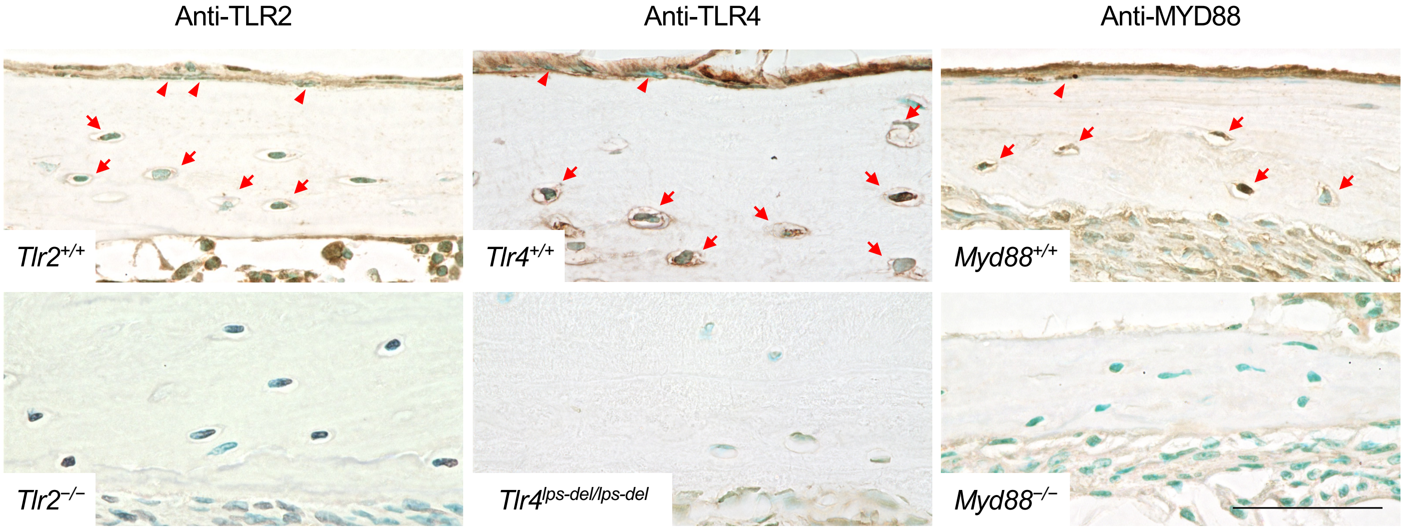

Supplementary Fig. 1: Osteocytes express TLR2, TLR4, and MYD88. Immunohistochemical staining of the calvarial bone tissue from 10-week-old mice using anti-TLR2, TLR4, and MYD88 antibodies. Arrows and arrowheads indicate osteocytes and osteoblasts, respectively, with positive staining. Representative images from three independent experiments with similar results. Scale bar = 50 μm.

Supplementary Figure 2

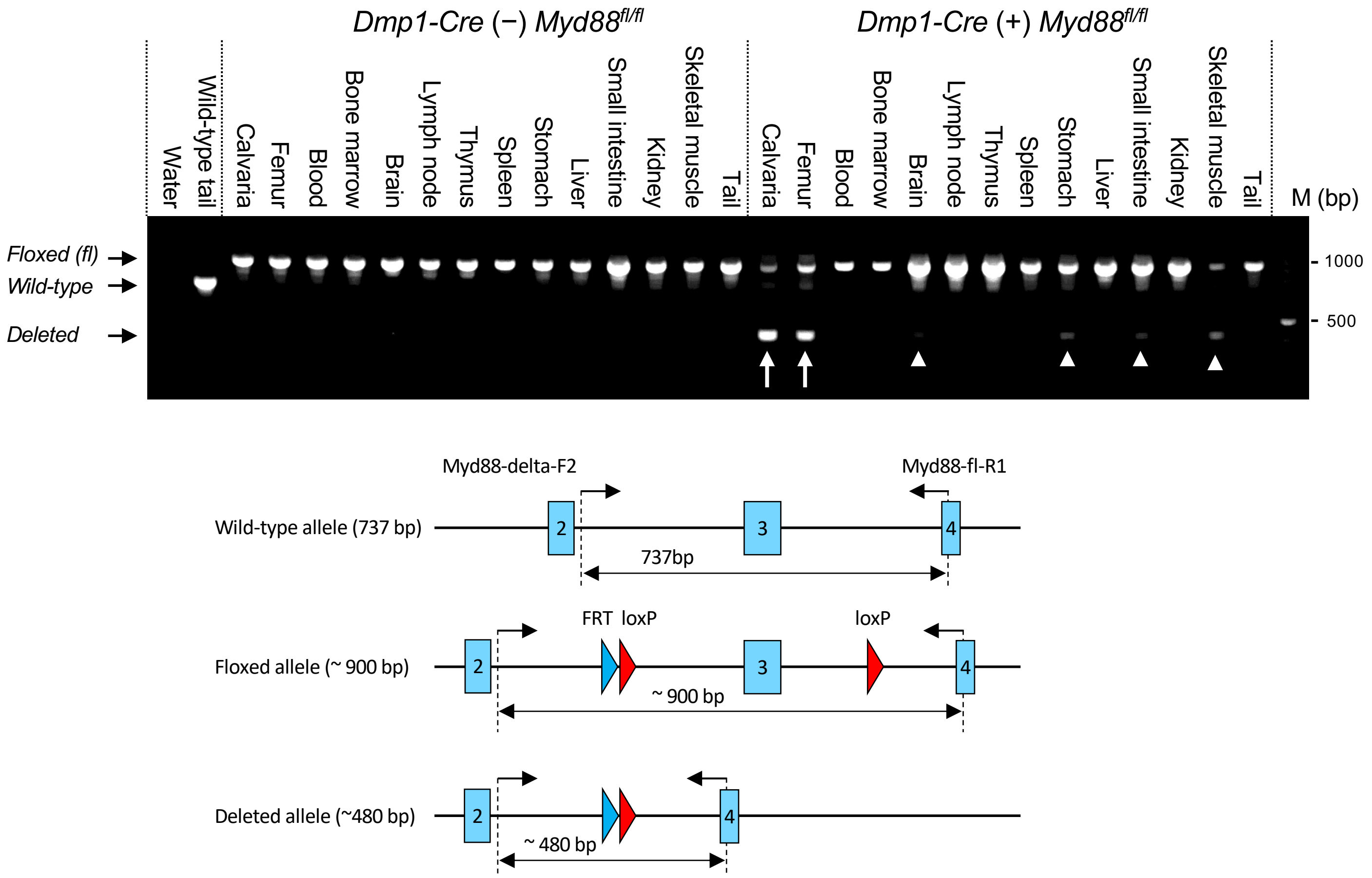

Supplementary Fig. 2: *Dmp1-Cre* does not cause *Myd88* gene recombination in immune and hematopoietic tissues*.* Confirmation of the lack of *Myd88* exon 3 deletion in immune and hematopoietic tissues in *Dmp1-Cre;Myd88*$^{fl/fl}$ mice by genomic PCR. Arrows indicate the predominant *Myd88* exon 3 deletion in the bone tissue. Arrowheads indicate the small off-target recombination in the brain, stomach, small intestine, and skeletal muscle (quadriceps) consistent with a report by Lim J et al., Bone Res 2017, 5, 16049. Diagrams represent the gene structure of each allele with primer locations. Primer sequences are listed in the Supplementary Table 4. Representative results from three independent experiments with similar results.

Supplementary Figure 3

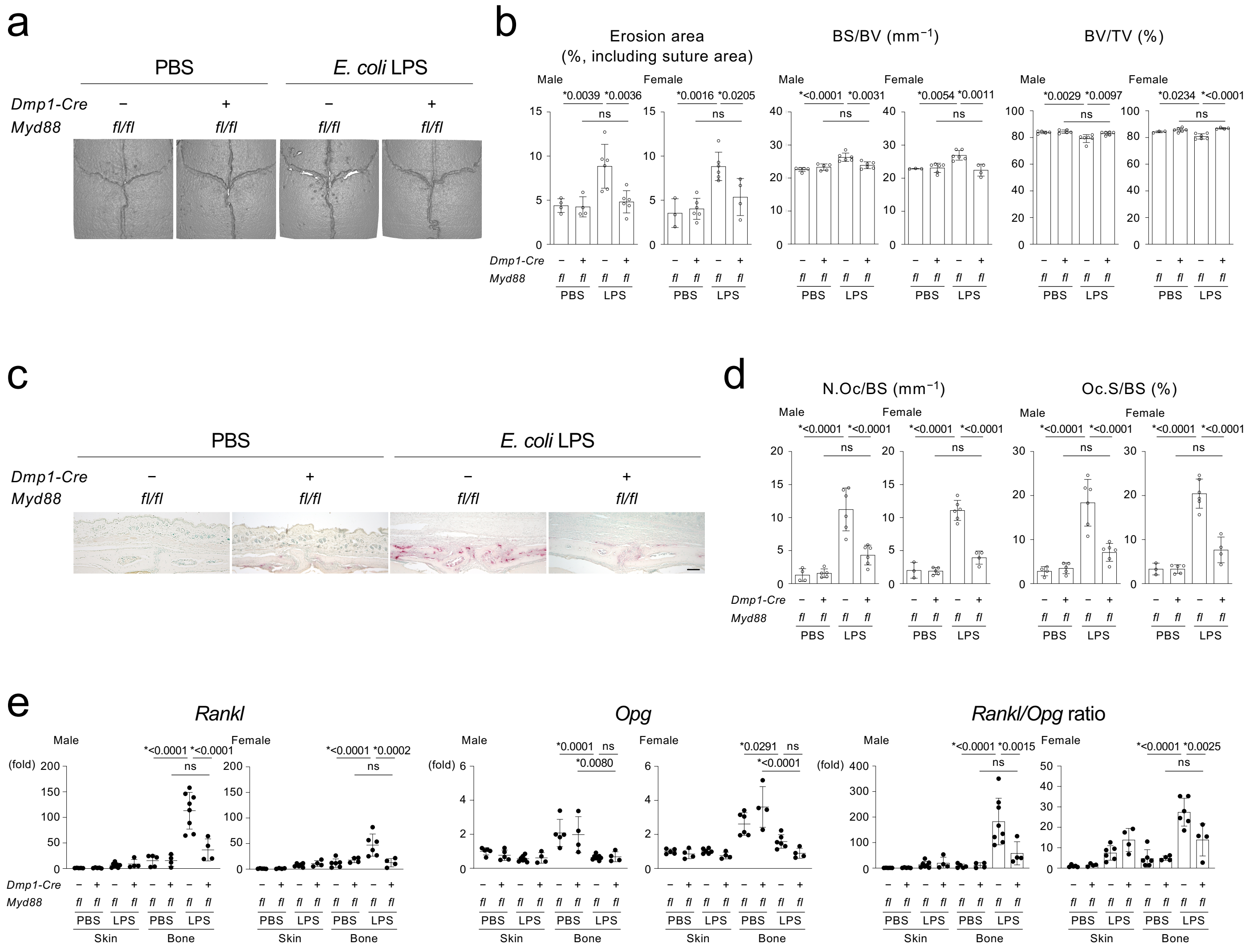

Supplementary Fig. 3: Lack of MYD88 in osteocytes and mature osteoblasts protects against calvarial osteolysis caused by *Escherichia coli* LPS. (a) MicroCT images of the calvaria from 10 to 11-week-old male mice with *Escherichia coli* (*E. coli*) LPS or PBS injection. Representative images from each group of male mice in (b) (n ≥ 4/group). **(b)** Erosion area, BS/BV, and BV/TV of the calvaria. **(c)** TRAP staining of the calvarial bone. Representative images from each group of male mice in (d) (n ≥ 4/group). Scale bar = 100 μm. **(d)** Histomorphometric analysis of osteoclasts on the calvarial bone surface at the intersection of the coronal and sagittal sutures. **(e)** qPCR analysis of *Rankl*, *Opg*, and *Rankl/Opg* in skin and calvarial bone tissues. **(b, d, e)** Data are presented as mean ± SD. *$p < 0.05$ with one-way ANOVA with Tukey-Kramer post-hoc test. ns = not significant. Each data point represents a biologically independent mouse. The number of data points represents the sample size (n) per group. Source data are provided as a Source Data file.

Sample size (n/group, from the left): **(b)** Erosion area (4, 4, 6, 6 in male; 3, 6, 6, 4 in female), BS/BV (5, 5, 6, 6 in male; 3, 6, 6, 4 in female), BV/TV (5, 5, 6, 6 in male; 3, 6, 6, 4 in female) **(d)** N.Oc/BS (4, 5, 6, 6 in male; 3, 5, 6, 4 in female), Oc.S/BS (4, 5, 6, 6 in male; 3, 5, 6, 4 in female) **(e)** *Rankl* (5, 5, 7, 4, 5, 4, 8, 4 in male; 5, 4, 6, 4, 6, 4, 6, 4 in female), *Opg* (5, 5, 7, 4, 5, 4, 8, 4 in male; 5, 4, 6, 4, 5, 4, 6, 4 in female), *Rankl/Opg* (5, 5, 7, 4, 5, 4, 8, 4 in male; 5, 4, 6, 4, 6, 4, 6, 4 in female).

Supplementary Figure 4

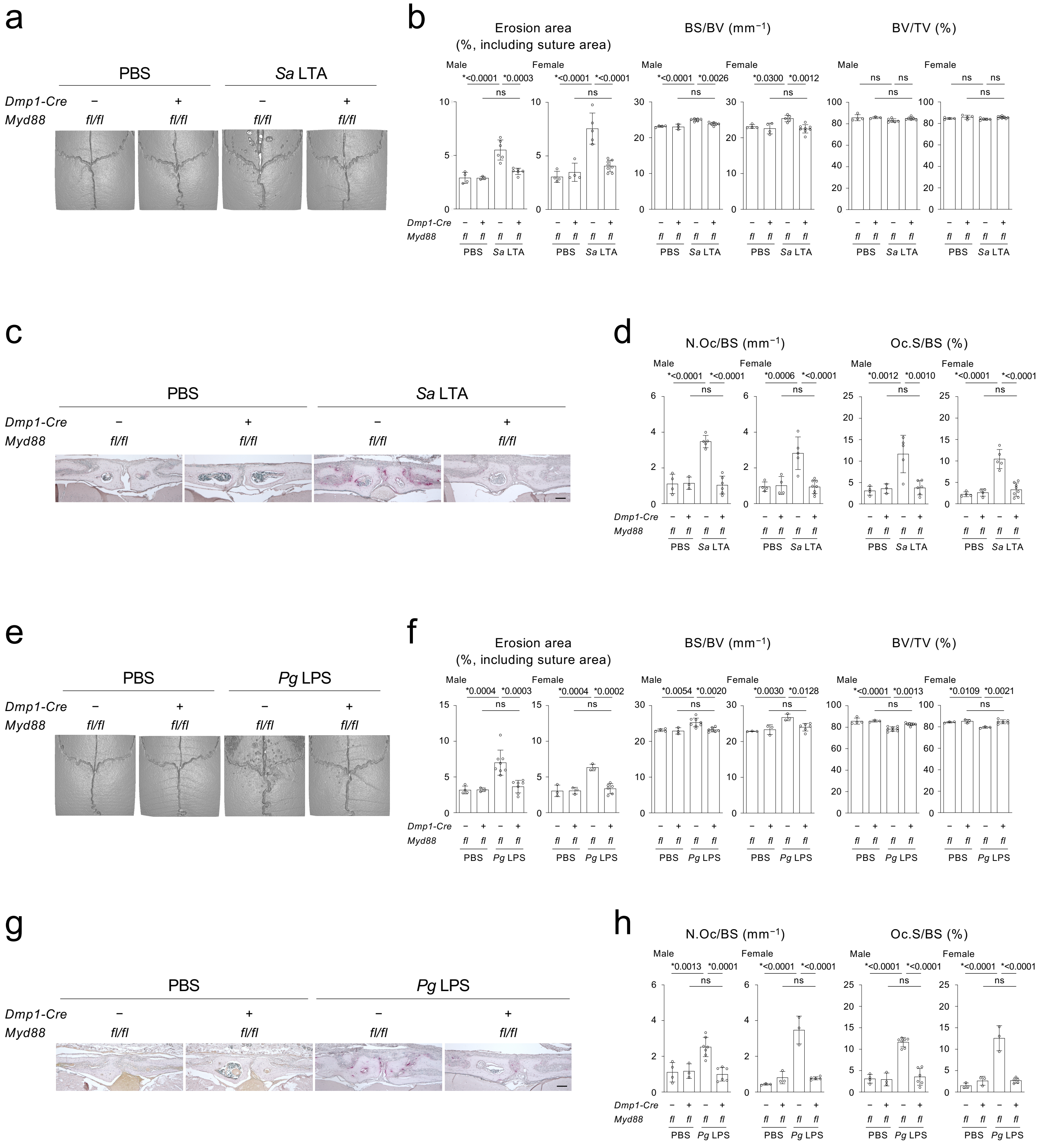

Supplementary Fig. 4: Lack of MYD88 in osteocytes and mature osteoblasts protects against calvarial osteolysis caused by *Staphylococcus aureus* LTA and *Porphyromonas gingivalis* LPS. (a, e) MicroCT images of the calvaria from 10 to 11-week-old male mice with *Staphylococcus aureus* (*Sa*) LTA, *Porphyromonas gingivalis* (*Pg*) LPS, or PBS injection. Representative images from each group of male mice in (b) ($n \geq 3$/group) and (f) ($n \geq 3$/group), respectively. **(b, f)** Erosion area, BS/BV, and BV/TV of the calvaria. **(c, g)** TRAP staining of the calvarial bone. Representative images from each group of male mice in (d) ($n \geq 3$/group) and (h) ($n \geq 3$/group), respectively. Scale bar = 100 µm. **(d, h)** Histomorphometric analysis of osteoclasts on the calvarial bone surface at the intersection of the coronal and sagittal sutures. **(b, d, f, h)** Data are presented as mean ± SD. *$p < 0.05$ with one-way ANOVA with Tukey-Kramer post-hoc test. ns = not significant. Each data point represents a biologically independent mouse. The number of data points represents the sample size (n) per group. Source data are provided as a Source Data file.

Sample size (n/group, from the left): **(b)** Erosion area (4, 3, 6, 6 in male; 4, 4, 5, 8 in female), BS/BV (4, 3, 6, 6 in male; 4, 4, 5, 8 in female), BV/TV (4, 3, 6, 6 in male; 4, 4, 5, 8 in female) **(d)** N.Oc/BS (4, 3, 5, 6 in male; 4, 4, 5, 8 in female), Oc.S/BS (4, 3, 5, 6 in male; 4, 4, 5, 8 in female) **(f)** Erosion area (4, 3, 8, 7 in male; 3, 3, 3, 6 in female), BS/BV (4, 3, 8, 7 in male; 3, 3, 3, 6 in female), BV/TV (4, 3, 8, 7 in male; 3, 3, 3, 6 in female) **(h)** N.Oc/BS (4, 3, 7, 6 in male; 3, 3, 3, 5 in female), Oc.S/BS (4, 3, 7, 6 in male; 3, 3, 3, 5 in female).

Supplementary Figure 5

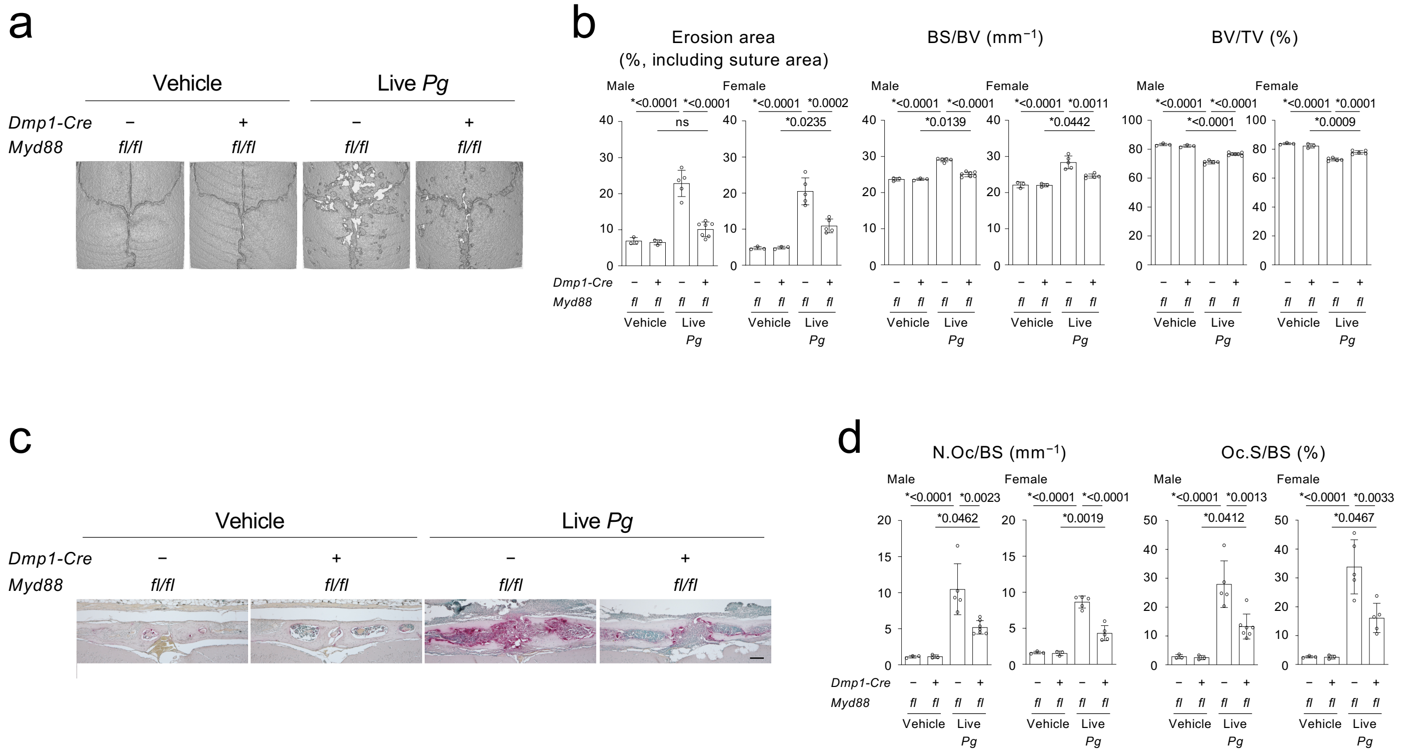

Supplementary Fig. 5: Lack of MYD88 in osteocytes and mature osteoblasts protects against calvarial osteolysis caused by live *Porphyromonas gingivalis*. (a) MicroCT images of the calvaria from 10 to 11-week-old male mice with live *Porphyromonas gingivalis* (*Pg*) or vehicle (culture medium) injection. Representative images from each group of male mice in (b) (n ≥ 3/group). **(b)** Erosion area, BS/BV, and BV/TV of the calvaria. **(c)** TRAP staining of the calvarial bone. Representative images from each group of male mice in (d) (n ≥ 3/group). Scale bar = 100 µm. **(d)** Histomorphometric analysis of osteoclasts on the calvarial bone surface at the intersection of the coronal and sagittal sutures. **(b, d)** Data are presented as mean ± SD. *$p < 0.05$ with one-way ANOVA with Tukey-Kramer post-hoc test. ns = not significant. Each data point represents a biologically independent mouse. The number of data points represents the sample size (n) per group. Source data are provided as a Source Data file.

Sample size (n/group, from the left): **(b)** Erosion area (3, 3, 5, 7 in male; 3, 3, 5, 5 in female), BS/BV (3, 3, 5, 7 in male; 3, 3, 5, 5 in female), BV/TV (3, 3, 5, 7 in male; 3, 3, 5, 5 in female) **(d)** N.Oc/BS (3, 3, 5, 7 in male; 3, 3, 5, 5 in female), Oc.S/BS (3, 3, 5, 7 in male; 3, 3, 5, 5 in female).

Supplementary Figure 6

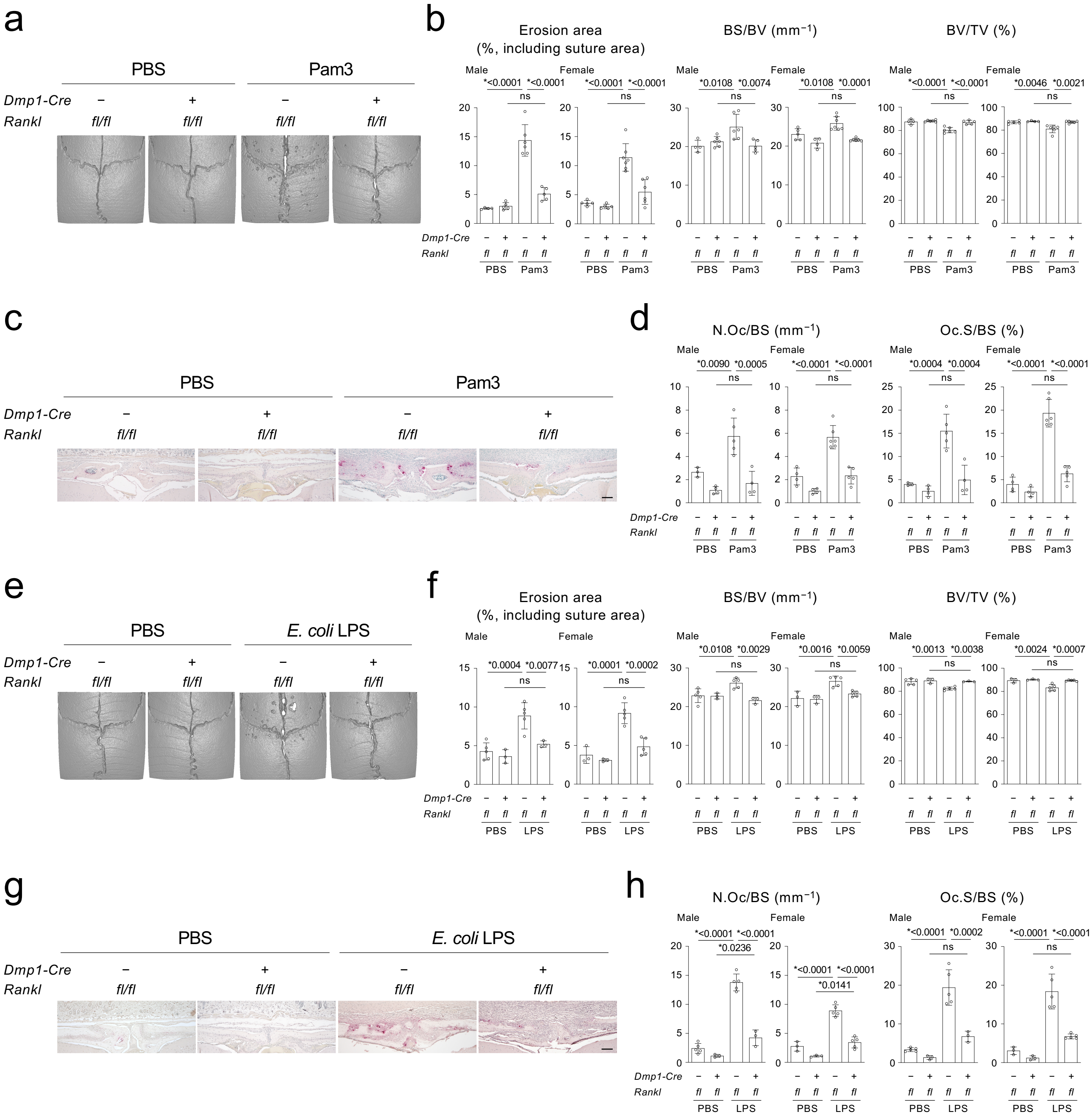

Supplementary Fig. 6: Lack of RANKL in osteocytes and mature osteoblasts protects against calvarial osteolysis caused by Pam3CSK4 and *Escherichia coli* LPS. (a, e) MicroCT images of the calvaria from 10 to 11-week-old male mice with Pam3CSK4 (Pam3), *Escherichia coli* (*E. coli*) LPS, or PBS injection. Representative images from each group of male mice in (b) (n ≥ 4/group) and (f) (n ≥ 3/group), respectively. **(b, f)** Erosion area, BS/BV, and BV/TV of the calvaria. **(c, g)** TRAP staining of the calvarial bone. Representative images from each group of male mice in (d) (n ≥ 3/group) and (h) (n ≥ 3/group), respectively. Scale bar = 100 µm. **(d, h)** Histomorphometric analysis of osteoclasts on the calvarial bone surface at the intersection of the coronal and sagittal sutures. **(b, d, f, h)** Data are presented as mean ± SD. *$p < 0.05$ with ANOVA with Tukey-Kramer post-hoc test. ns = not significant. Each data point represents a biologically independent mouse. The number of data points represents the sample size (n) per group. Source data are provided as a Source Data file.

Sample size (n/group, from the left): **(b)** Erosion area (4, 5, 6, 5 in male; 5, 5, 7, 6 in female), BS/BV (4, 6, 6, 5 in male; 5, 4, 7, 6 in female), BV/TV (4, 6, 6, 5 in male; 5, 4, 7, 6 in female) **(d)** N.Oc/BS (3, 4, 5, 4 in male; 4, 4, 6, 5 in female), Oc.S/BS (3, 4, 5, 4 in male; 4, 4, 6, 5 in female) **(f)** Erosion area (5, 3, 5, 3 in male; 3, 3, 5, 5 in female), BS/BV (5, 3, 5, 3 in male; 3, 3, 5, 5 in female), BV/TV (5, 3, 5, 3 in male; 3, 3, 5, 5 in female) **(h)** N.Oc/BS (5, 3, 5, 3 in male; 3, 3, 5, 5 in female), Oc.S/BS (5, 3, 5, 3 in male; 3, 3, 5, 5 in female).

Supplementary Figure 7

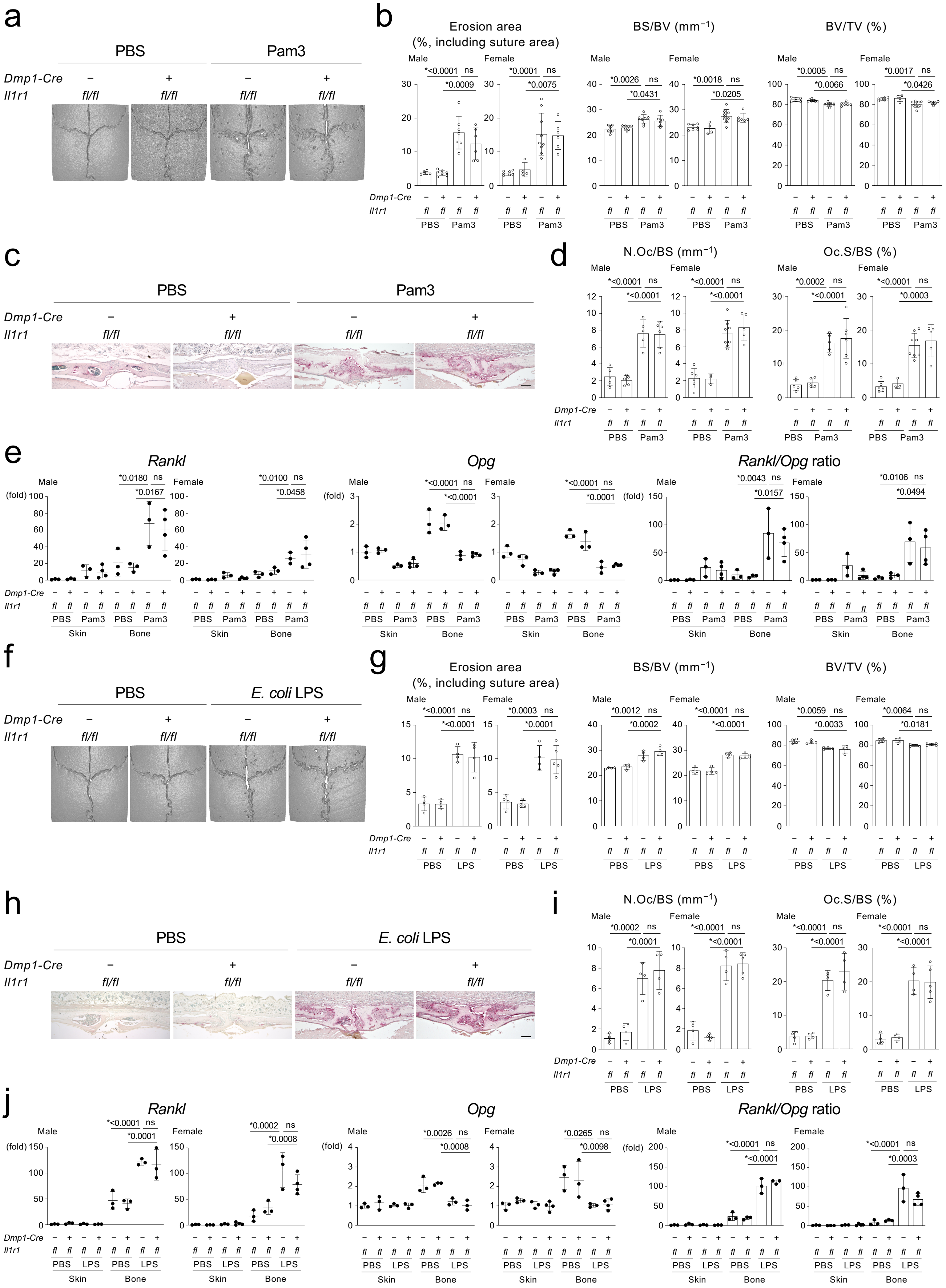

Supplementary Fig. 7: Lack of IL-1R in osteocytes and mature osteoblasts fails to rescue calvarial osteolysis caused by Pam3CSK4 and *Escherichia coli* LPS. (a, f) MicroCT images of the calvaria from 10 to 11-week-old male mice with Pam3CSK4 (Pam3), *Escherichia coli* (*E. coli*) LPS, or PBS injection. Representative images from each group of male mice in (b) (n ≥ 6/group) and (g) (n = 4/group), respectively. **(b, g)** Erosion area, BS/BV, and BV/TV of the calvaria. **(c, h)** TRAP staining of the calvarial bone. Representative images from each group of male mice in (d) (n ≥ 5/group) and (i) (n = 4/group), respectively. Scale bar = 100 μm. **(d, i)** Histomorphometric analysis of osteoclasts on the calvarial bone surface at the intersection of the coronal and sagittal sutures. **(e, j)** qPCR analysis of *Rankl*, *Opg*, and *Rankl/Opg* in skin and calvarial bone tissues. **(b, d, e, g, i, j)** Data are presented as mean ± SD. **p* < 0.05 with ANOVA with Tukey-Kramer post-hoc test. ns = not significant. Each data point represents a biologically independent mouse. The number of data points represents the sample size (n) per group. Source data are provided as a Source Data file.

Sample size (n/group, from the left): **(b)** Erosion area (6, 7, 7, 6 in male; 7, 4, 9, 6 in female), BS/BV (6, 7, 7, 6 in male; 7, 4, 9, 6 in female), BV/TV (6, 7, 7, 6 in male; 7, 4, 9, 6 in female) **(d)** N.Oc/BS (5, 5, 5, 6 in male; 6, 3, 8, 5 in female), Oc.S/BS (5, 5, 5, 6 in male; 6, 3, 8, 5 in female) **(e)** *Rankl* (3, 3, 3, 4, 3, 3, 3, 4 in male; 3, 3, 3, 4, 3, 3, 3, 4 in female), *Opg* (3, 3, 3, 4, 3, 3, 3, 4 in male; 3, 3, 3, 4, 3, 3, 3, 4 in female), *Rankl/Opg* (3, 3, 3, 4, 3, 3, 3, 4 in male; 3, 3, 3, 4, 3, 3, 3, 4 in female) **(g)** Erosion area (4, 4, 4, 4 in male; 4, 4, 4, 5 in female), BS/BV (4, 4, 4, 4 in male; 4, 4, 4, 5 in female), BV/TV (4, 4, 4, 4 in male; 4, 4, 4, 5 in female) **(i)** N.Oc/BS (4, 4, 4, 4 in male; 4, 4, 4, 5 in female), Oc.S/BS (4, 4, 4, 4 in male; 4, 4, 4, 5 in female) **(j)** *Rankl* (3, 3, 3, 3, 3, 3, 3, 3 in male; 3, 3, 3, 4, 3, 3, 3, 4 in female), *Opg* (3, 3, 3, 3, 3, 3, 3, 3 in male; 3, 3, 3, 4, 3, 3, 3, 4 in female), *Rankl/Opg* (3, 3, 3, 3, 3, 3, 3, 3 in male; 3, 3, 3, 4, 3, 3, 3, 4 in female).

Supplementary Figure 8

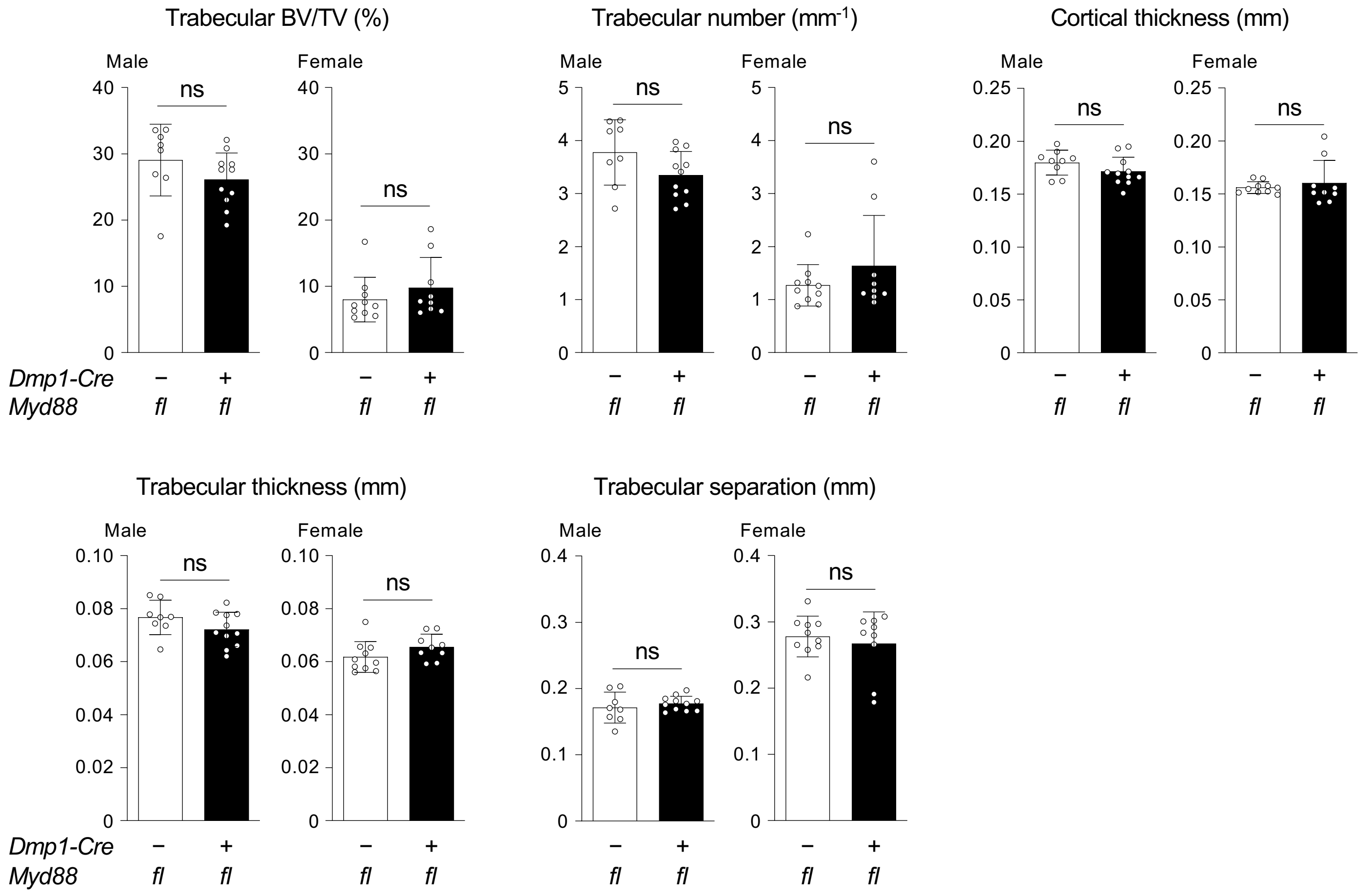

Supplementary Fig. 8: Lack of MYD88 in osteocytes and mature osteoblasts does not affect bone mass under physiological conditions. MicroCT analysis of the femur from 10 to 11 weeks old mice. Data are presented as mean ± SD. Two-tailed unpaired t-test. ns = not significant. Each data point represents a biologically independent mouse. The number of data points represents the sample size (n) per group. Source data are provided as a Source Data file.

Sample size (n/group, from the left): Trabecular BV/TV (8, 11 in male; 10, 9 in female), Trabecular number (8, 11 in male; 10, 9 in female), Cortical thickness (9, 11 in male; 10, 9 in female), Trabecular thickness (8, 11 in male; 10, 9 in female), Trabecular separation (8, 11 in male; 10, 9 in female).

Supplementary Figure 9

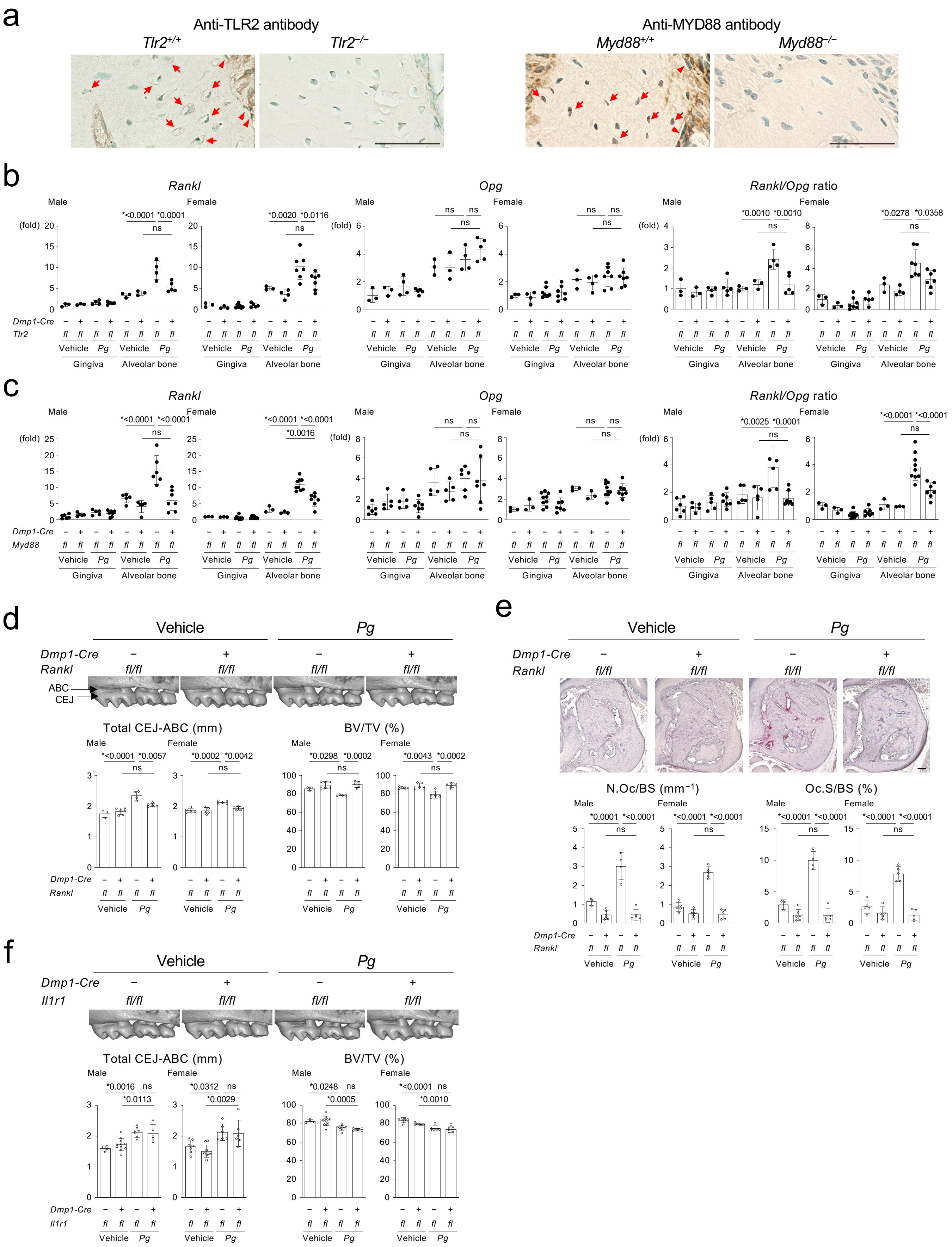

Supplementary Fig. 9: Impact of the TLR2-MYD88 axis, RANKL, and IL-1R in osteocytes and mature osteoblasts on *Porphyromonas gingivalis*-induced alveolar bone loss. (a) Immunohistochemical analysis of TLR2 and MYD88 in alveolar bone tissue from 10-week-old male mice. Arrows and arrowheads indicate osteocytes and osteoblasts, respectively, with positive staining. Representative images from three independent experiments. Scale bar = 50 μm. **(b, c)** qPCR analysis of *Rankl*, *Opg*, and *Rankl/Opg* in the gingiva and alveolar bone from mice inoculated with *Porphyromonas gingivalis* (*Pg*) or vehicle. **(d, f)** Top: MicroCT images of the right maxilla from male mice inoculated with *Pg* or vehicle. Buccal side view. Representative images from each group of male mice (n ≥ 3/group). Bottom: The total CEJ-ABC distance of the right maxillary molars and alveolar BV/TV underneath the second molar of the right maxilla. **(e)** Top: TRAP staining of the alveolar bone. Representative images from each group of male mice (n ≥ 3/group). Scale bar = 100 μm. Bottom: Histomorphometric analysis of osteoclasts on the alveolar bone surface. **(b−f)** Data are presented as mean ± SD. **p* < 0.05 with ANOVA with Tukey-Kramer post-hoc test. ns = not significant. Each data point represents a biologically independent mouse. The number of data points represents the sample size (n) per group. Source data are provided as a Source Data file.

Sample size (n/group, from the left): **(b)** *Rankl* (3, 3, 4, 5, 3, 3, 4, 5 in male; 3, 3, 7, 5, 3, 4, 7, 7 in female), *Opg* (3, 3, 4, 5, 3, 3, 4, 5 in male; 3, 4, 7, 7, 3, 4, 7, 7 in female), *Rankl/Opg* (3, 3, 4, 5, 3, 3, 4, 5 in male; 3, 3, 7, 5, 3, 4, 7, 7 in female) **(c)** *Rankl* (6, 5, 5, 7, 5, 5, 6, 7 in male; 3, 3, 9, 7, 3, 3, 9, 7 in female), *Opg* (6, 5, 5, 7, 5, 5, 6, 7 in male; 3, 3, 9, 7, 3, 3, 9, 7 in female), *Rankl/Opg* (6, 5, 5, 7, 5, 5, 6, 7 in male; 3, 3, 9, 7, 3, 3, 9, 7 in female) **(d)** Total CEJ-ABC (3, 6, 4, 5 in male; 5, 5, 5, 5 in female), BV/TV (3, 6, 4, 5 in male; 5, 5, 5, 5 in female) **(e)** N.Oc/BS (3, 6, 4, 5 in male; 5, 5, 5, 5 in female), Oc.S/BS (3, 6, 4, 5 in male; 5, 5, 5, 5 in female) **(f)** Total CEJ-ABC (4, 11, 7, 5 in male; 7, 9, 6, 6 in female), BV/TV (4, 11, 8, 5 in male; 6, 9, 6, 6 in female).

Supplementary Figure 10

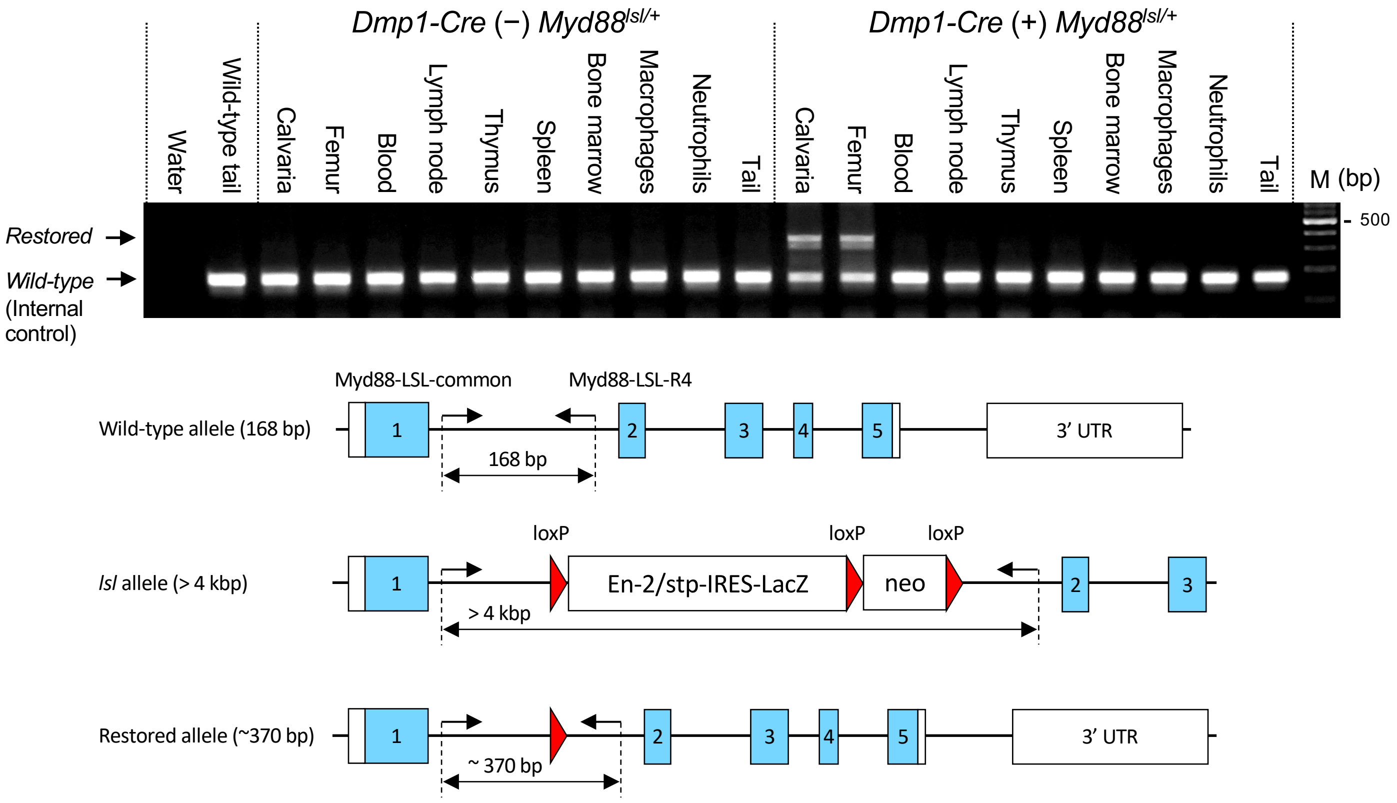

Supplementary Fig. 10: *Dmp1-Cre* does not cause *Myd88* gene restoration in immune cells and tissues. Confirmation of the lack of *Myd88* restoration in immune cells and tissues in *Dmp1-Cre;Myd8*$^{lsl/+}$ mice by genomic PCR. Diagrams represent the gene structure of each allele with primer locations. Bone marrow-derived M-CSF-dependent macrophages were used as macrophages. Neutrophils were isolated from the bone marrow using the MojoSort mouse neutrophil isolation kit. Primer sequences are listed in the Supplementary Table 4. Representative results from three independent experiments with similar results.

Supplementary Figure 11

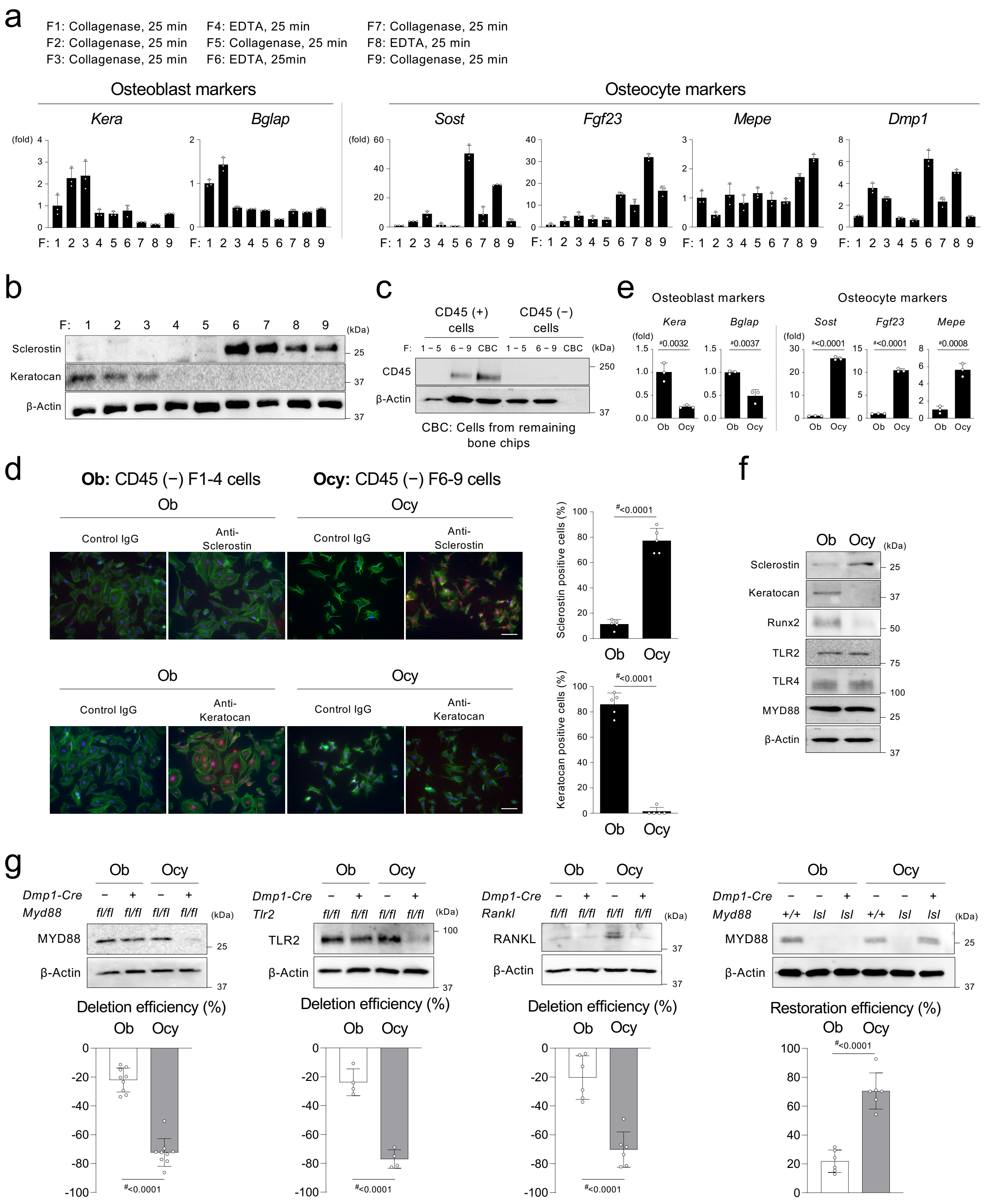

Supplementary Fig. 11: Validation of the procedure for differential isolation of the osteocyte-enriched and osteoblast-enriched cell populations from adult mouse calvariae. (a) qPCR analysis of osteoblast and osteocyte marker gene expression in each cell fraction enzymatically isolated from calvarial bones of 10 to 11-week-old C57BL/6J male mice. Calvariae from fifteen mice were pooled and serially digested with collagenase and EDTA. Each fraction was cultured on dishes coated with rat tail collagen and harvested after 48 hours. F = fraction. Average expression levels in F1 were set as 1. Data are representative of five independent experiments with similar results, each with three replicates (n = 3). **(b)** Western blotting of sclerostin (encoded by *Sost*) and keratocan (encoded by *Kera*) using cell lysates from each calvarial cell fraction. **(c)** Western blotting of CD45 to confirm the elimination of hematopoietic cells from pooled F1-5 and F-6-9 cells. F6-9 fractions contained significantly more hematopoietic cells than F1-5 fractions. Cells from remaining bone chip (CBC) were mainly consist of hematopoietic cells. **(d)** Immunocytochemical staining of the Ob (CD45 (−) F1-4 cells) and Ocy (CD45 (−) F6-9 cells) with anti-sclerostin or keratocan antibody (red), confirming the enrichment of osteocytes in Ocy and osteoblasts in Ob. F5 was excluded to better separate Ocy from Ob. Representative images from five independent experiments. Graphs show the percentage of sclerostin- or keratocan-positive cells using the image data from five independent experiments (n = 5). Blue: DAPI. Green: F-actin visualized by phalloidin staining. Scale bar = 100 μm. **(e)** qPCR analysis of osteoblast and osteocyte marker genes in Ob (pooled F1−4 depleted for CD45+ cells) and Ocy (pooled F6−9 depleted for CD45+ cells). Average expression levels in Ob were set as 1. Data are representative of three independent experiments, each with three replicates (n = 3). **(f)** Western blotting of sclerostin, keratocan, Runx2, TLR2, TLR4, MYD88, and β-Actin in Ob and Ocy, confirming the differential keratocan and RUNX2 expression in Ob and sclerostin expression in Ocy. **(g)** Western blotting of MYD88, TLR2, and RANKL using cell lysates of Ob and Ocy isolated from *Dmp1-Cre;Myd88*$^{fl/fl}$, *Dmp1-Cre;Tlr2*$^{fl/fl}$, *Dmp1-Cre;Rankl*$^{fl/fl}$, *Dmp1-Cre;Myd88*$^{lsl/lsl}$, and their control mice. Graphs show the deletion or restoration efficiency measured by Image J. Each data point represents an independent isolation of Ob and Ocy from mice with the indicated genotypes. *Myd88*$^{fl/fl}$ and *Dmp1-Cre;Myd88*$^{fl/fl}$ mice (n = 9 times), *Tlr2*$^{fl/fl}$ and *Dmp1-Cre;Tlr2*$^{fl/fl}$ mice (n = 4 times), *Rankl*$^{fl/fl}$ and *Dmp1-Cre;Rankl*$^{fl/fl}$ mice (n = 6 times), *Myd88*$^{lsl/lsl}$ and *Dmp1-Cre;Myd88*$^{lsl/lsl}$ (n = 6 times). **(b, c, f, g)** Representative images from more than three independent experiments with similar results. **(a, d, e, g)** Data are presented as mean ± SD. $^{\#}p < 0.05$ with two-tailed unpaired t-test. Source data are provided as a Source Data file.

Supplementary Figure 12

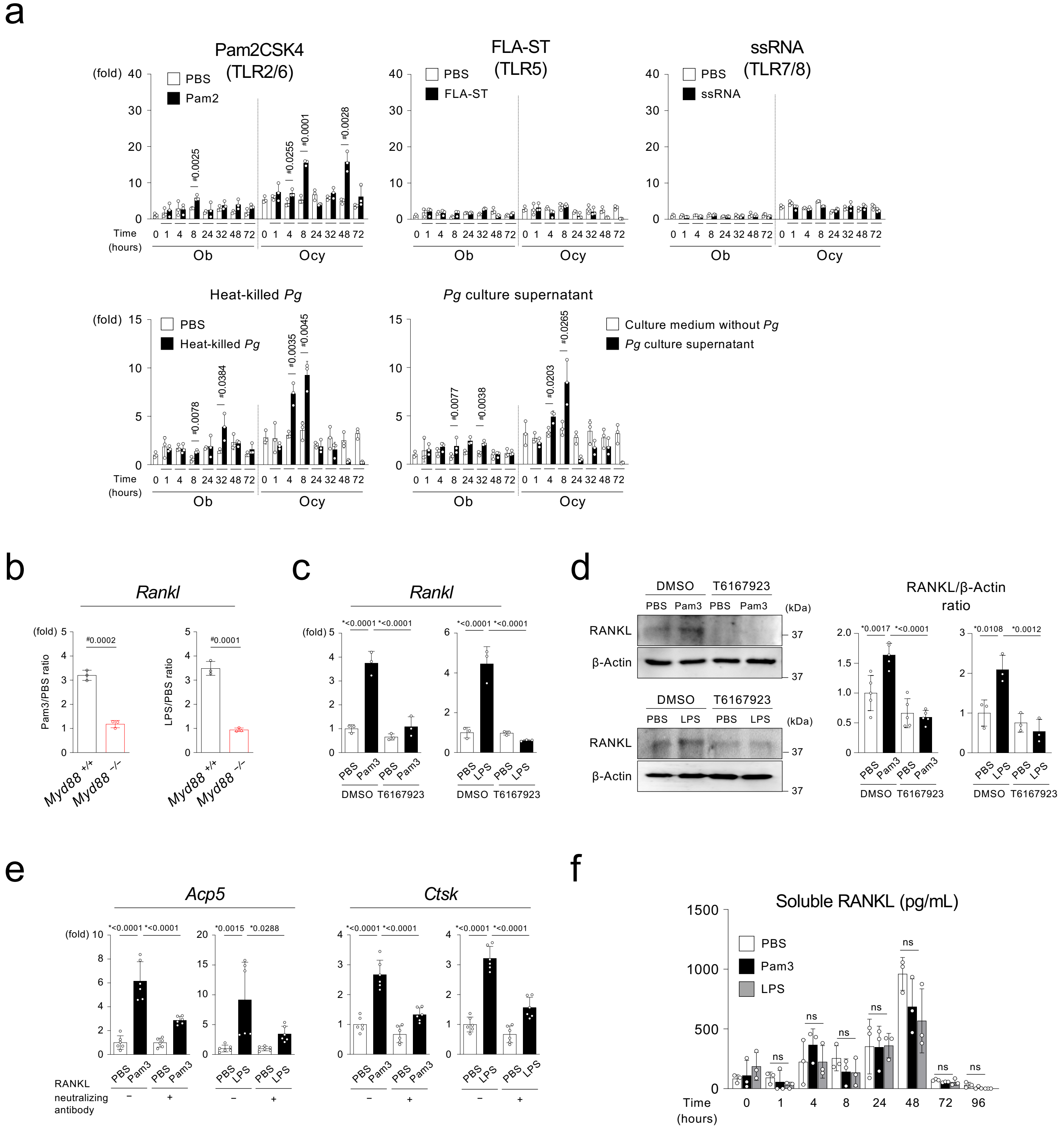

Supplementary Fig. 12: Direct regulation of RANKL expression via the TLR2/4-MYD88 pathway in the osteocyte-enriched cell population. (a) qPCR analysis of *Rankl* in Ob and Ocy stimulated with Pam2CSK4 (Pam2, 100 ng/mL), FLA-ST (100 ng/mL), single stranded RNA (ssRNA, 100 ng/mL), heat-killed *Porphyromonas gingivalis* (*Pg*) (MOI: 10), or *Pg* culture supernatant every 24 hours. Average expression levels in Ob treated with PBS for 0 hour were set as 1. Representative data from three independent experiments with similar results, each with three replicates (n = 3). **(b)** qPCR analysis of *Rankl* in Ocy isolated from *Myd88*$^{-/-}$ and *Myd88*$^{+/+}$ male mice. Ocy was stimulated with Pam3CSK4 (Pam3) or *E. coli* LPS for 8 hours. Data are presented as fold induction against PBS. Data are from three independent experiments (n = 3). **(c)** qPCR analysis of *Rankl* in Ocy isolated from wild-type C57BL/6J mice. Ocy was stimulated with Pam3 or *E. coli* LPS for 8 hours with or without pretreatment of T6167923 (20 μM). T6167923 was added in cultures 2 hours before Pam3 or *E. coli* LPS stimulation. Average *Rankl* expressions when treated with DMSO/PBS were set as 1. Representative data from three independent experiments with similar results, each with three replicates (n = 3). **(d)** Left: Western blotting of RANKL in Ocy stimulated with Pam3 or *E. coli* LPS with or without pretreatment of T6167923 (20 μM). Results at 48 hours after Pam3 or *E. coli* LPS stimulation. Right: Relative RANKL protein levels. Averages of relative intensities against β-Actin when treated with DMSO/PBS were set as 1. Representative images from five (Pam3) and three (LPS) independent experiments with similar results. Data in graphs are from five (Pam3) and three (LPS) independent experiments. **(e)** qPCR analysis of *Acp5* and *Ctsk*. RNAs were isolated from co-cultures of Ocy with bone marrow-derived M-CSF-dependent macrophages from *Myd88*$^{-/-}$ mice stimulated with Pam3 or *E. coli* LPS in the presence or absence of RANKL neutralizing antibody (125 ng/mL). Average expression levels in co-cultures stimulated PBS in the absence of RANKL neutralizing antibody were set as 1. Representative data from three independent experiments with similar results, each with six replicates (n = 6). **(f)** ELISA for soluble RANKL in Ocy culture supernatants. Ocy was stimulated with Pam3 or *E. coli* LPS every 24 hours. Data are representative of two independent experiments (n = 3/group). **(a−f)** Data are presented as mean ± SD. $^{\#}p < 0.05$ with two-tailed unpaired t-test (a, b). $^{*}p < 0.05$ with one-way ANOVA with Tukey-Kramer post-hoc test (c, d, e, f). ns = not significant. Source data are provided as a Source Data file.

Supplementary Figure 13

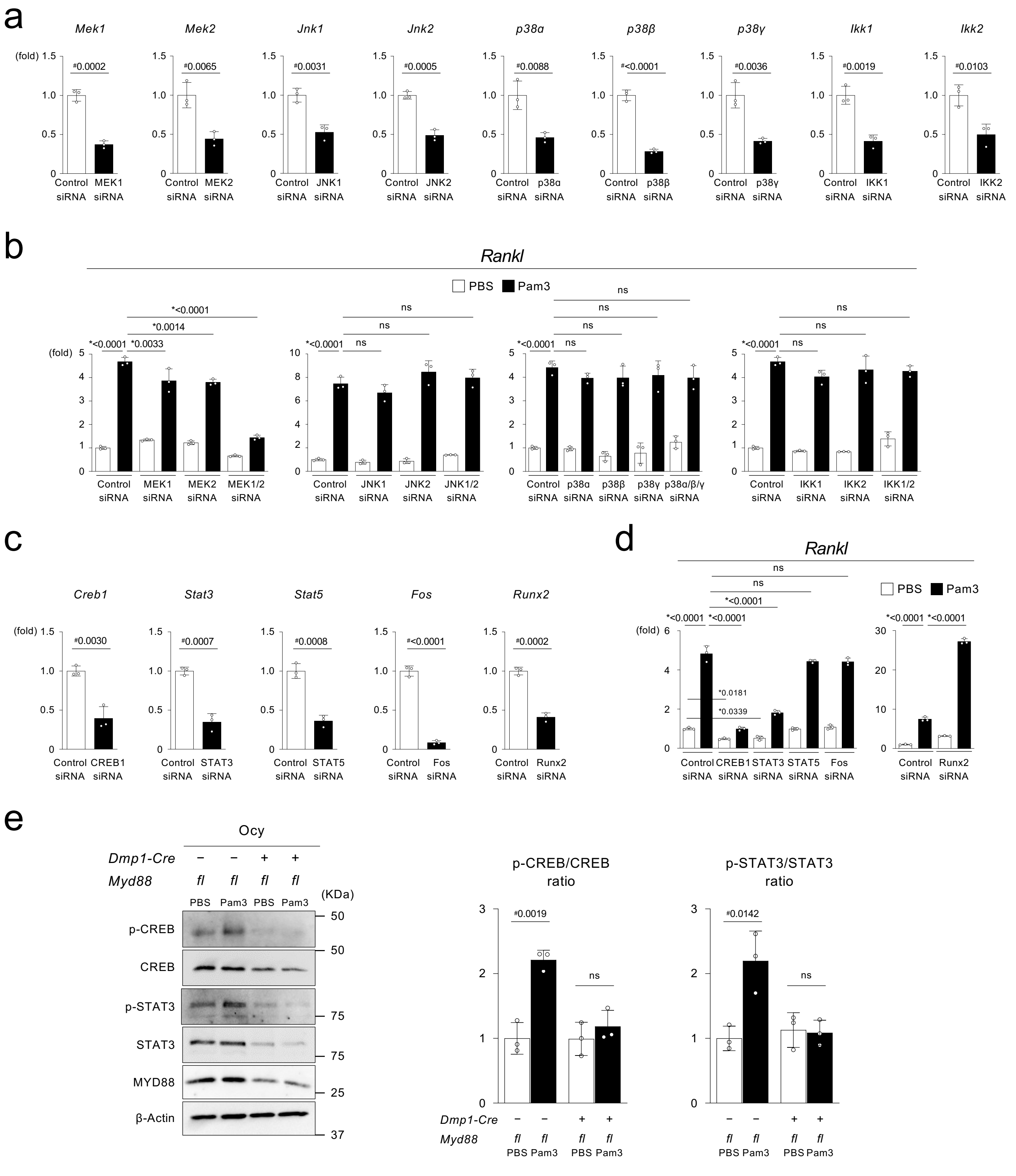

Supplementary Fig. 13: CREB- and STAT3-mediated *Rankl* induction in MLO-Y4 cells stimulated with Pam3CSK4 and lack of CREB and STAT3 activation in osteocyte-enriched cells deficient in MYD88. (a, c) Confirmation of siRNA-mediated knockdown of (a) *Mek1*, *Mek2*, *Jnk1*, *Jnk3*, *p38α*, *p38β*, *p38γ*, *Ikk1*, or *Ikk2* and (c) *Creb1*, *Stat3*, *Stat5*, *Fos*, or *Runx2* in MLO-Y4 cells by qPCR. 48 hours after siRNA transfection. Results from a single experiment with three replicates (n = 3). Average expression levels in MLO-Y4 cells transfected with control siRNA were set as 1. **(b, d)** qPCR analysis of *Rankl* in MLO-Y4 cells stimulated Pam3CSK4 (Pam3) or PBS for 8 hours. Results from a single experiment with three replicates (n = 3). Average expression levels when treated with control siRNA/PBS were set as 1. **(e)** Left: Western blotting of indicated proteins in cell lysates from Ocy. Ocy was isolated from $Myd88^{fl/fl}$ and $Dmp1\text{-}Cre;Myd88^{fl/fl}$ mice, then stimulated with Pam3 or PBS for 8 hours. Representative images from three independent experiments. Right: Densitometric analysis of the p-CREB/CREB and p-STAT3/STAT3 ratio using ImageJ. Averages of relative intensities in $Myd88^{fl/fl}$ Ocy treated with PBS were set as 1. Data are from three independent experiments (n = 3). **(a−e)** Data are presented as mean ± SD. $^{\#}p < 0.05$ with two-tailed unpaired t-test (a, c, e). $^{*}p < 0.05$ with one-way ANOVA with Tukey-Kramer post-hoc test (b, d). ns = not significant. Source data are provided as a Source Data file.

Supplementary Figure 14

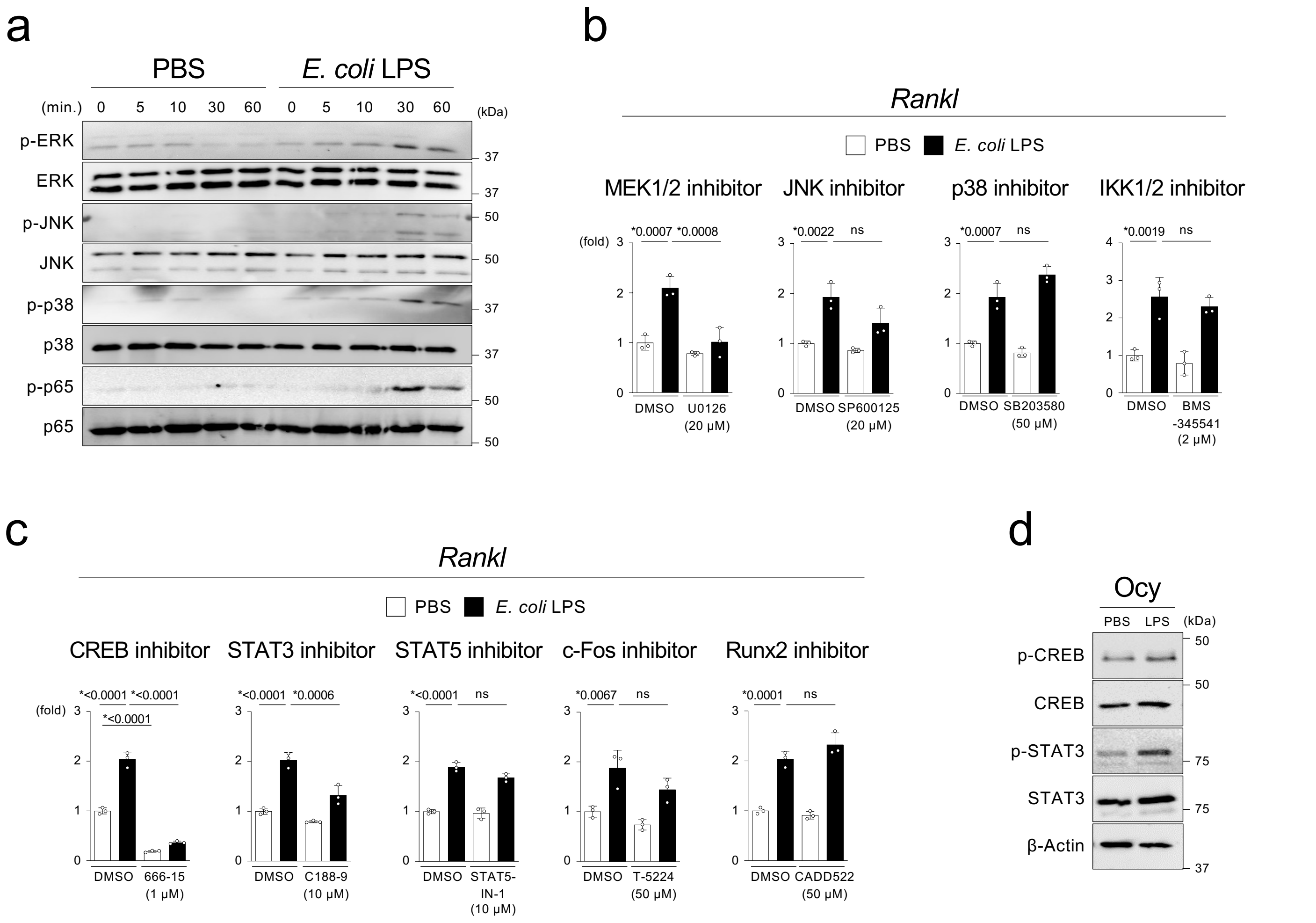

Supplementary Fig. 14: CREB and STAT3 mediate *Rankl* induction in osteocyte-enriched cells stimulated with *Escherichia coli* LPS. (a) Western blotting of phosphorylated (p) and total ERK, JNK, p38, and NF-kB p65 proteins using cell lysates from Ocy stimulated with *Escherichia coli* (*E. coli*) LPS. Representative images from three independent experiments. **(b, c)** qPCR analysis of *Rankl* in Ocy. Ocy was pretreated with inhibitors or vehicle (DMSO) for 2 hours before stimulation with *E. coli* LPS or PBS for 8 hours. Average expression levels in Ocy treated with DMSO/PBS were set as 1. Representative data from three independent experiments with similar results, each with three replicates (n = 3). **(d)** Western blotting of phosphorylated (p) and total CREB and STAT3 proteins using cell lysates from Ocy stimulated with *E. coli* LPS for 8 hours. Representative images from three independent experiments with similar results. **(b, c)** Data are presented as mean ± SD. *$p < 0.05$ with one-way ANOVA with Tukey-Kramer post-hoc test. ns = not significant. Source data are provided as a Source Data file.

Supplementary Figure 15

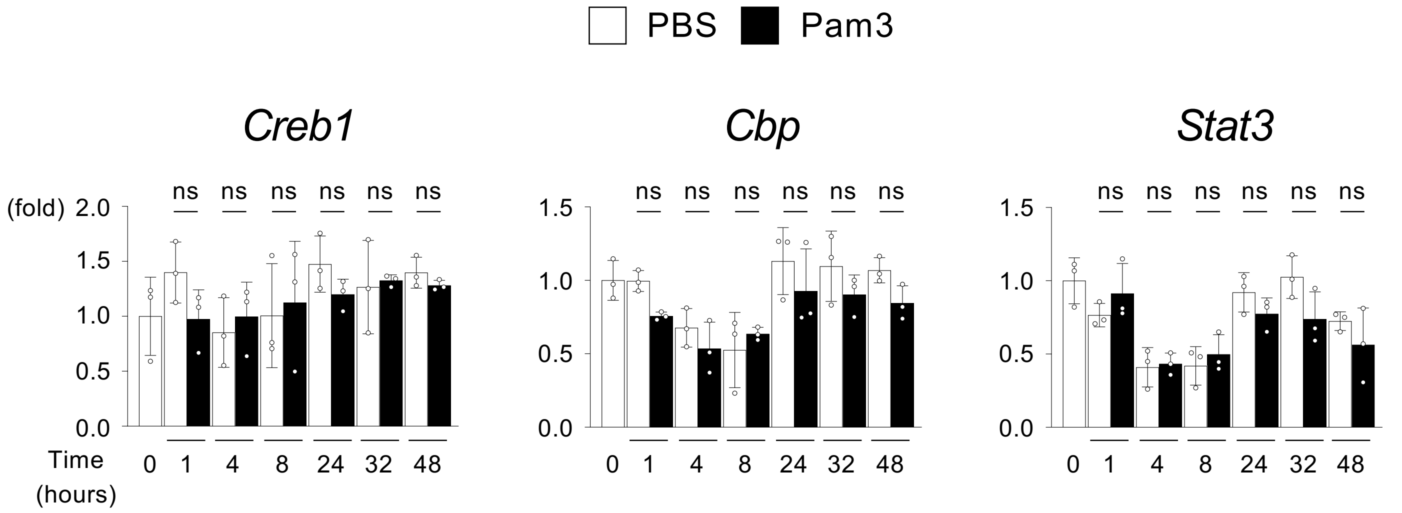

Supplementary Fig. 15: Activation of the MYD88 pathway does not affect *Creb1*, *Cbp*, or *Stat3* levels in osteocyte-enriched cells. qPCR analysis of *Creb1*, *Cbp*, and *Stat3* in Ocy stimulated with Pam3CSK4 (Pam3). Data are representative of three independent experiments with similar results, each with three replicates (n = 3). Data are presented as mean ± SD. Two-tailed unpaired t-test. ns = not significant. Source data are provided as a Source Data file.

Supplementary Figure 16

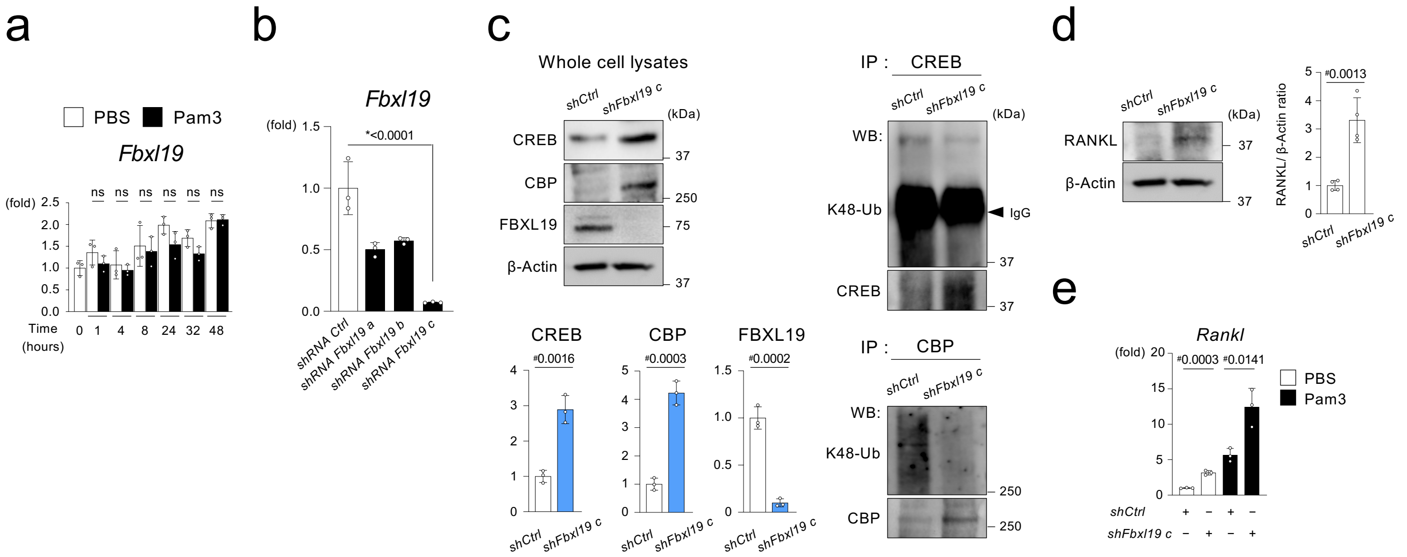

Supplementary Fig. 16: FBXL19 is a ubiquitin ligase for CREB and CBP in osteocytes to regulate RANKL expression. (a) qPCR analysis of *Fbxl19* in Ocy stimulated with Pam3CSK4 (Pam3). Data are representative of three independent experiments with similar results, each with three replicates (n = 3). **(b)** qPCR analysis to determine the knockdown efficiency of *Fbxl19* in undifferentiated IDG-SW3 cells. Three different lentiviruses (a, b, c) expressing the different short hairpin RNA (shRNA) were individually infected. Results from a single experiment with three replicates (n = 3). **(c)** Left: Western blotting of the indicated proteins and graphs showing the relative CREB, CBP, and FBXL19 protein levels normalized by β-Actin. Whole cell lysates of differentiated IDG-SW3 cells were used. Data in graphs are from three independent experiments. Right: Western blotting of K48-ubiquitinated proteins after immunoprecipitation (IP) of CREB or CBP. IDG-SW3 cells were differentiated to osteocytic cells and used for IP. **(d)** Left: Western blotting of RANKL in differentiated IDG-SW3 cells. Right: Relative RANKL protein level normalized by β-Actin. Data in the graph are from four independent experiments. **(e)** qPCR analysis of *Rankl* in differentiated IDG-SW3 cells stimulated with Pam3 or PBS for 48 hours. Data are representative of three independent experiments with similar results, each with three replicates (n = 3). **(a−e)** Data are presented as mean ± SD. $^{\#}p < 0.05$ with two-tailed unpaired t-test (a, c, d, e). $^{*}p < 0.05$ with one-way ANOVA with Tukey-Kramer post-hoc test (b). ns = not significant. **(b−e)** Ctrl = control. **(c, d)** Representative images from three and four independent experiments with similar results, respectively. Source data are provided as a Source Data file.

Supplementary Figure 17

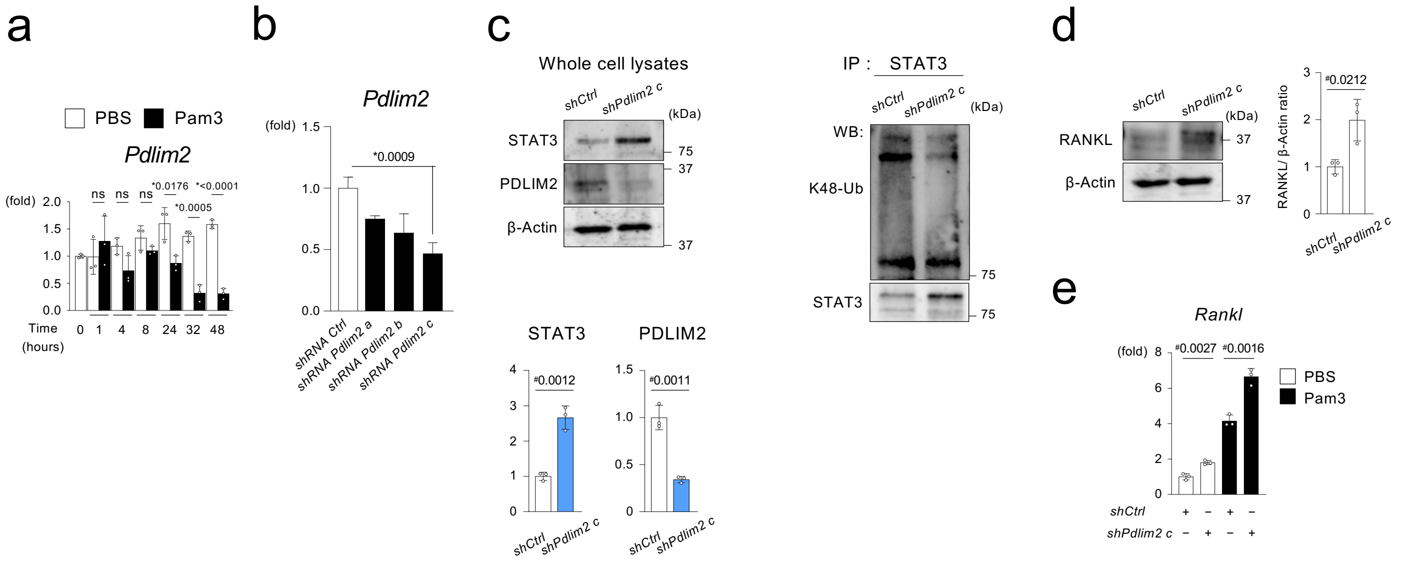

Supplementary Fig. 17: PDLIM2 is a ubiquitin ligase for STAT3 in osteocytes to regulate RANKL expression. (a) qPCR analysis of *Pdlim2* in Ocy stimulated with Pam3CSK4 (Pam3). Data are representative of three independent experiments with similar results, each with three replicates (n = 3). **(b)** qPCR to determine the knockdown efficiency of *Pdlim2* in undifferentiated IDG-SW3 cells. Three different lentiviruses (a, b, c) expressing the different short hairpin RNA (shRNA) were individually infected. Results from a single experiment with three replicates (n = 3). **(c)** Left: Western blotting of the indicated proteins and graphs showing the relative STAT3 and PDLIM2 protein levels normalized by β-Actin. Whole cell lysates of differentiated IDG-SW3 cells were used. Data in graphs are from three independent experiments. Right: Western blotting of K48-ubiquitinated proteins after immunoprecipitation (IP) of STAT3. IDG-SW3 cells were differentiated to osteocytic cells and used for IP. **(d)** Left: Western blotting of RANKL in differentiated IDG-SW3 cells. Right: Relative RANKL protein level normalized by β-Actin. Data in the graph are from three independent experiments. **(e)** qPCR analysis of *Rankl* in differentiated IDG-SW3 cells stimulated with Pam3 or PBS for 48 hours. Data are representative of three independent experiments with similar results, each with three replicates (n = 3). **(a−e)** Data are presented as mean ± SD. $^{\#}p < 0.05$ with two-tailed unpaired t-test (a, c, d, e). $^{*}p < 0.05$ with one-way ANOVA with Tukey-Kramer post-hoc test (b). ns = not significant. **(b−e)** Ctrl = control. **(c, d)** Representative images from three independent experiments with similar results. Source data are provided as a Source Data file.

Supplementary Figure 18

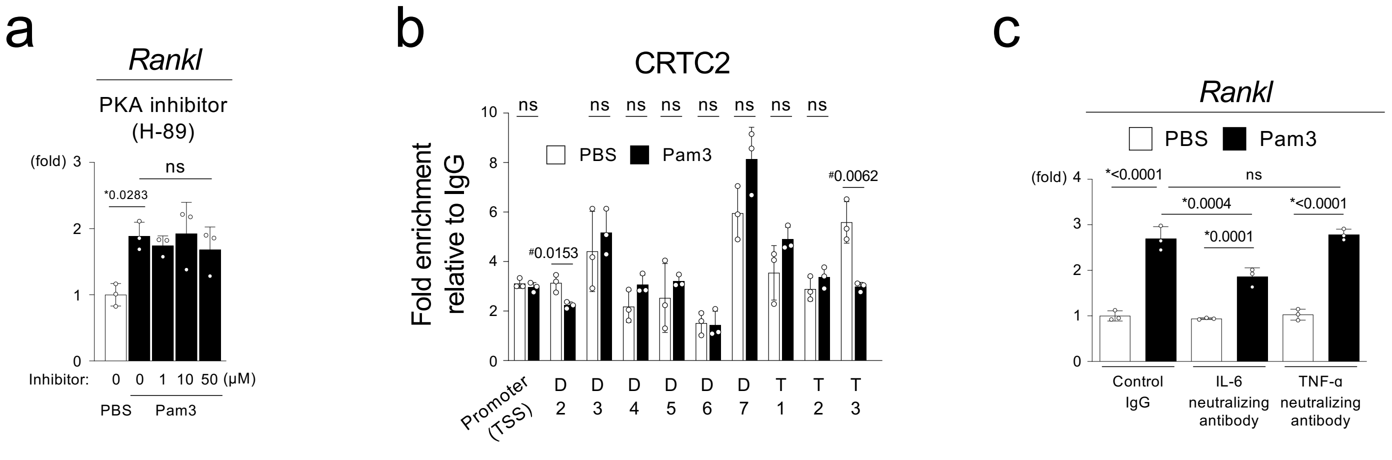

Supplementary Fig. 18: The PKA-CRTC2 axis is not involved in the mechanism of MYD88-mediated *Rankl* induction and IL-6 acts in an autocrine or paracrine manner to promote *Rankl* expression in osteocyte-enriched cells stimulated with Pam3CSK4. (a) qPCR analysis of *Rankl* in Ocy. Ocy was pretreated with H-89 or vehicle (DMSO) for 2 hours before stimulation with Pam3CSK4 (Pam3) or PBS for 8 hours. **(b)** CUT & RUN assays of CRTC2 in Ocy stimulated with Pam3 or PBS for 8 hours. Inductions of CRTC2 binding to the *Rankl* promoter and enhancers were quantitated by qPCR relative to the isotype control IgG (fold enrichment). **(c)** qPCR analysis of *Rankl* in Ocy stimulated with Pam3 or PBS in the presence of IL-6 or TNF-α neutralizing antibodies or control IgG. Ocy was pretreated with neutralizing antibodies for 3 hours, then stimulated with Pam3 or PBS for 48 hours. **(a−c)** Data are representative of three independent experiments with similar results, each with three replicates (n = 3) and presented as mean ± SD. $^{*}p < 0.05$ with one-way ANOVA with Tukey-Kramer post-hoc test (a, c). $^{\#}p < 0.05$ with two-tailed unpaired t-test (b). ns = not significant. Source data are provided as a Source Data file.

Supplementary Figure 19

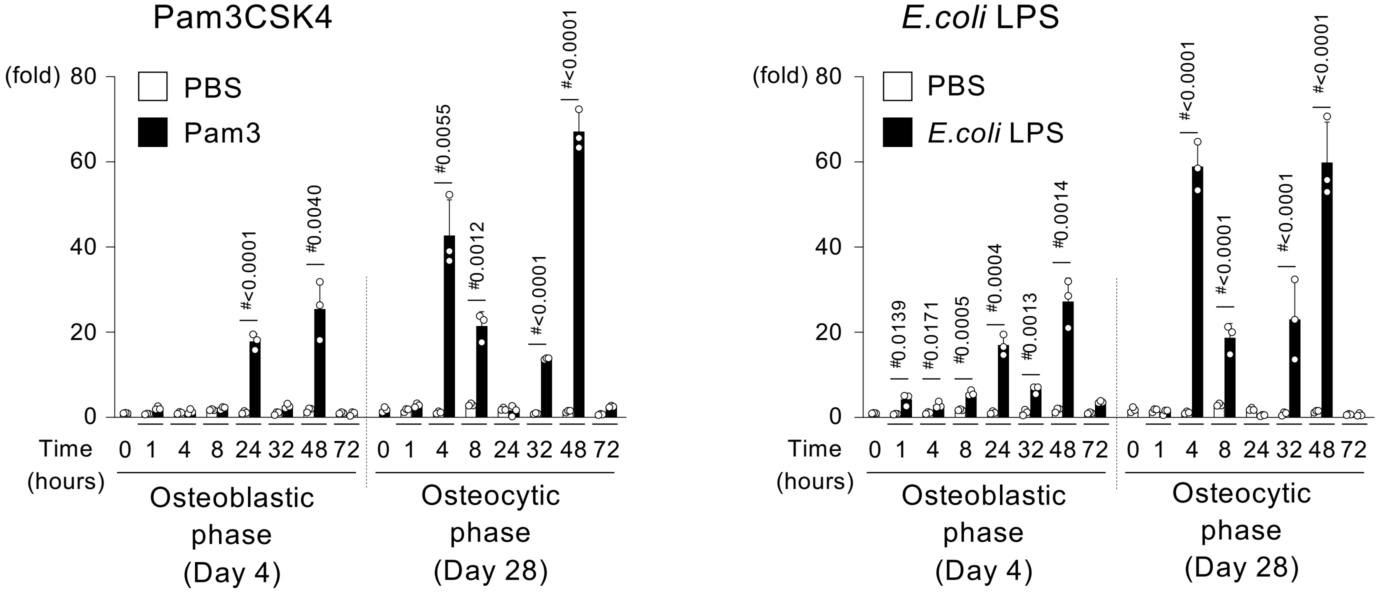

Supplementary Fig. 19: Bacterial PAMPs induce *Rankl* more rapidly and robustly in osteocytic IDG-SW3 cells than osteoblastic IDG-SW3 cells. qPCR analysis of *Rankl*. IDG-SW3 cells in the osteoblastic phase (day 4) and osteocytic phase (day 28) were stimulated with Pam3CSK4 (Pam3), *Escherichia coli* (*E. coli*) LPS, or PBS every 24 hours. Average expression levels in osteoblastic IDG-SW3 cells treated with PBS for 0 hour were set as 1. Data are representative of three independent experiments with similar results, each with three replicates (n = 3) and presented as mean ± SD. $^{\#}p < 0.05$ with two-tailed unpaired t-test. Source data are provided as a Source Data file.

Supplementary Figure 20

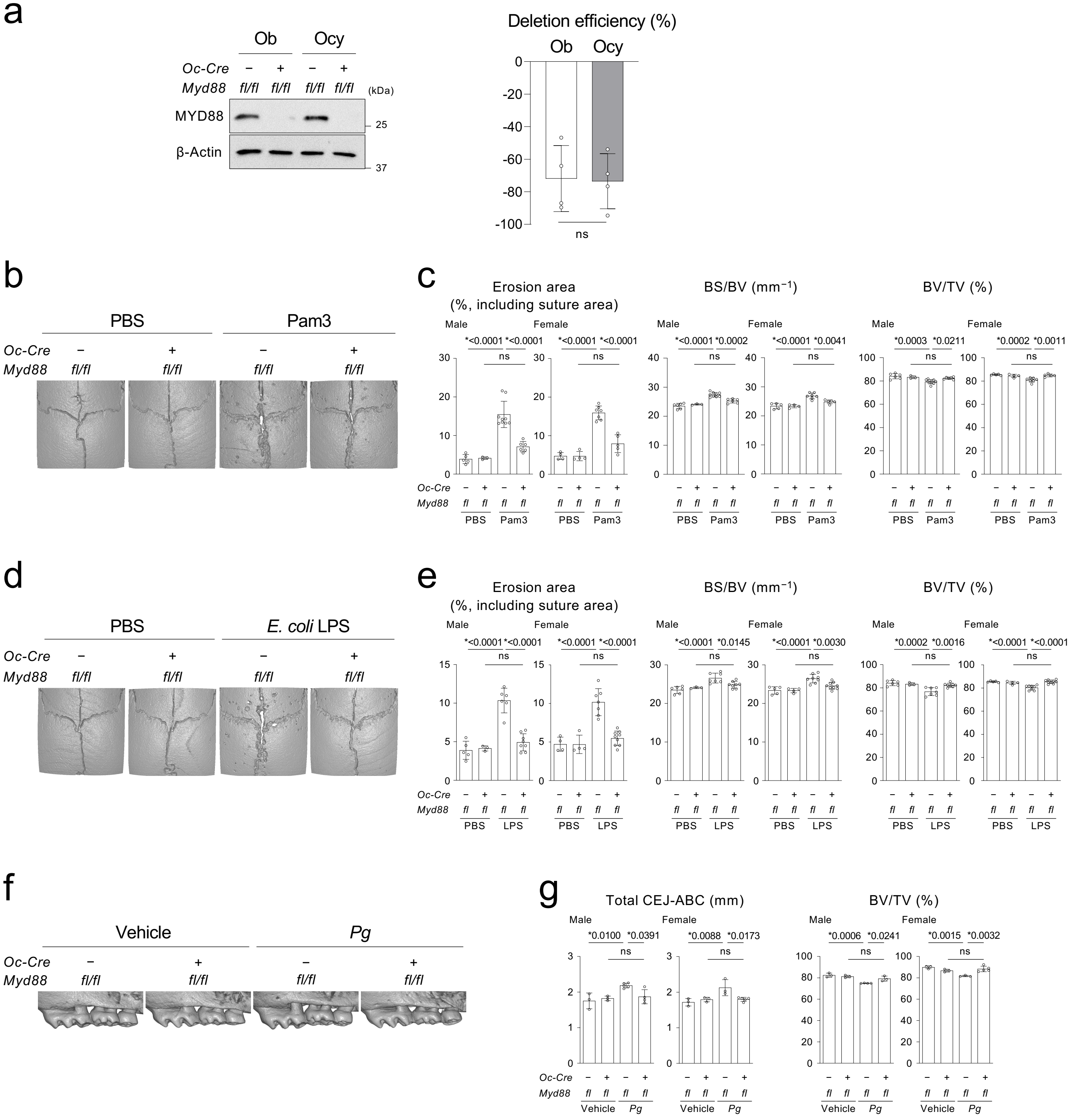

Supplementary Fig. 20: Deletion of MYD88 by *Osteocalcin-Cre* rescues calvarial osteolysis caused by PAMPs and alveolar bone loss induced by oral *Porphyromonas gingivalis* infection. (a) Western blotting of MYD88 using cell lysates of Ob and Ocy isolated from *Osteocalcin (Oc)-Cre;Myd88*$^{fl/fl}$ and *Myd88*$^{fl/fl}$ mice. Representative images from four independent experiments. The graph shows the deletion efficiency measured by Image J. Each data point represents an independent isolation of Ob and Ocy from mice with the indicated genotypes (n = 4 times). **(b, d)** MicroCT images of the calvaria from 10 to 11-week-old male mice with Pam3CSK4 (Pam3), *Escherichia coli* (*E. coli*) LPS or PBS injection. Representative images from each group of male mice in (c) (n ≥ 3/group) and (e) (n ≥ 3/group), respectively. **(c, e)** Erosion area, BS/BV, and BV/TV of the calvaria. **(d, e)** LPS data are at day 5 after two-time LPS injections on days 1 and 3. PBS data are at day 7 after three-time PBS injections on days 1, 3, and 5. The same PBS-control values as (c) were used in (e) because *E. coli* LPS injection was started at the same time as Pam3 and PBS injection. **(f)** MicroCT images of the right maxilla from male mice inoculated with *Pg* or vehicle. Buccal side view. Representative images from each group of male mice in (g) (n ≥ 3/group). **(g)** The total CEJ-ABC distance of the right maxillary molars and alveolar BV/TV underneath the second molar of the right maxilla. **(a)** Data are presented as mean ± SD. ns = not significant with two-tailed unpaired t-test. **(c, e, g)** Data are presented as mean ± SD. *$p < 0.05$ with one-way ANOVA with Tukey-Kramer post-hoc test. ns = not significant. Each data point represents a biologically independent mouse. The number of data points represents the sample size (n) per group. Source data are provided as a Source Data file.

Sample size (n/group, from the left): **(c)** Erosion area (5, 3, 9, 6 in male; 4, 4, 7, 5 in female), BS/BV (6, 3, 9, 6 in male; 5, 4, 7, 5 in female), BV/TV (6, 3, 9, 6 in male; 5, 4, 7, 5 in female) **(e)** Erosion area (5, 3, 6, 8 in male; 4, 4, 7, 9 in female), BS/BV (6, 3, 7, 8 in male; 5, 4, 8, 9 in female), BV/TV (6, 3, 7, 8 in male; 5, 4, 8, 9 in female) **(g)** Total CEJ-ABC (3, 3, 5, 4 in male; 3, 3, 3, 5 in female), BV/TV (3, 3, 4, 3 in male; 3, 3, 3, 5 in female).

Supplementary Figure 21

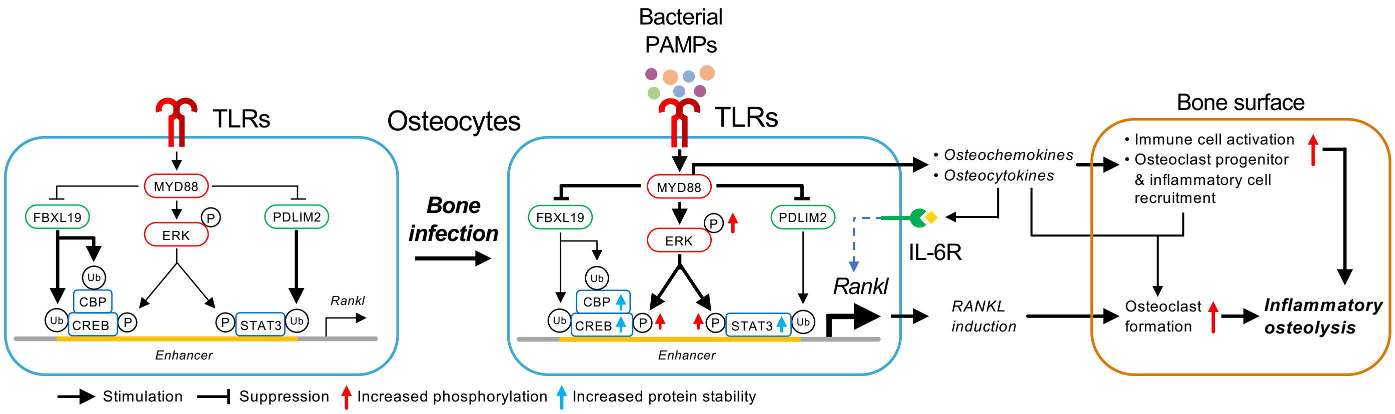

Supplementary Fig. 21: A schematic model of osteocyte regulation of inflammatory osteolysis via the TLR-MYD88 pathway in bone infection. Activation of the MYD88 pathway induces the phosphorylation of CREB and STAT3 via ERK and promotes the stabilization of CREB/CBP and STAT3 proteins by suppressing the ubiquitination of these TFs mediated by FBXL19 or PDLIM2. These molecular events increase the binding of CREB and STAT3 to RANKL enhancers, leading to increased RANKL expression responsible for osteoclast induction. IL-6 from osteocytes may contribute to boosting the RANKL expression. At the same time, the activated MYD88 pathway stimulates the expression of inflammatory mediators, inducing the immune cell activation on the bone surface and the recruitment/migration of osteoclast progenitor and inflammatory cells to the bone surface. Further investigation is needed to determine the mechanism by which the MYD88 pathway activation regulates the expression, degradation, and function of FBXL19 and PDLIM2 and their interaction with CREB/CBP and STAT3, respectively. Also, it remains to be elucidated whether and how CREB and STAT3 interact with each other within the same enhancer region or between distant enhancer regions to control *Rankl* transcription. The thickness of the lines in osteocytes represents the relative activity of the pathways between PAMPs-stimulated and non-stimulated osteocytes.

Supplementary Table 1: Primers for qPCR.

Gene	Forward (5'-3')	Reverse (5'-3')
Acp5	cagcagcccaaaatgcct	ttttgagccaggacagctga
Adgre1	tctggggagcttacgatgga	gaatcccgcaatgatggcac
β-Actin	ggctgtattcccctccatcg	ccagttggtaacaatgccatgt
Cbp	gaccgctttgtttatacctgc	tcttatgggtgtggctctttg
c-Fos	gggacagcctttcctactacc	gatctgcgcaaaagtcctgt
Creb1	cattgcccctggagttgttatg	tctacgacattctcttgctgcctc
Ctsk	cgaaaagagcctagcgaaca	tgggtagcagcagaaacttg
Dc-stamp	cgaagctccttgagaaacga	ggactggaaaccagaaatgaa
Dmp1	ggctgtcctgtgctctcccag	ggtcactatttgcctgtccctc
Fbxl19	gagggagaagacgagaagtttagtt	gttgggtatctcggaattgataac
Fgf23	agccatgactcgaaggttcc	gctcgcgagagcaggatac
Gapdh	atcaagaaggtggtgaagca	gacaacctggtcctcagtgt
Ikk1	ccgtgttctcaaggagctgt	tcacacatgtcagaggatgttca
Ikk2	cgttctgcagcaaggagaga	gtcaacggtcacggtgtact
Il1b	agttgacggaccccaaaag	agctggatgctctcatcagg
Il6	aacgatgatgcacttgcaga	ccagaggaaattttcaataggc
Jnk1	cttcagaagcagaagcccca	tgtgctaaaggagacggctg
Jnk2	agtgggttgcatcatgggag	gtgtgctcagtggacatgga
Kera	tcccccatcaacttattttagc	ggttgccattacaggacctt
Ly6g	ttgcaaagtcctgtgtgctc	aggggcaggtagttgtgttg
Mek1	cactttctccaagctggggc	agccataaccaggccagatg
Mek2	ctatggggccttctacagcg	gagcaccgcaatgctgac
Mepe	gtgaatgacgccagagggcctca	tgtcttgattgccgcagctgct
Mmp9	acggacccgaagcggac	gggatacccgtctccgtgc
Opg	tgtccagatgggttcttctca	cgttgtcatgtgttgcatttcc
p38α	cacagggacctaaagcccag	ttcttcagaagctcagcccc
p38β	tacctcgtgacgaccctgat	agctgagtagaggggacagg
p38γ	ctggtgtcatccacagagacttga	tgtagttcttggcctctgcac
Pdlim2	gagaacatgctacacgcgga	ggagccctggaatctggttg
Rankl	tgaagacacactacctgactcctg	ccacaatgtgttgcagttcc
Runx2	tgagatttgtgggccggag	gtgcctgcctgggatttcttg
Sost	tcctgagaagaaccagacca	gcagctgtactcggacacatc
Stat3	acctccaggacgactttgat	tgtcttctgcacgtactcca
Stat5	gcaccttcagatcaaccaaac	cagctgggcaaactgag
Tnf	gatcggtccccaaagggatg	tgtgagggtctgggccatag

Supplementary Table 2: Materials used in this study.

Reagent	Source	Identifier
**Antibody** (WB: Western Blotting, IF: Immunofluorescent staining, IHC: Immunohistochemistry)		
FBXL19 (EPR11957, 1:1000 for WB)	Abcam	Cat# ab172961; RRID: AB_2801257
Keratocan (1:500 for WB, 1:100 for IF)	Abcam	Cat# ab128304; RRID: AB_11144483
MYD88 (1:1000 for WB)	Abcam	Cat# ab135693; RRID: AB_2802168
TLR4 (1:100 for IHC)	Abcam	Cat# ab13867; RRID: AB_300696
F4/80 (CI:A3-1, 1:200 for IHC)	Bio-Rad	Cat# MCA497R, RRID:AB_323279
Anti-mouse IgG HRP-linked antibody (1:5000 for WB)	Cell Signaling Technology	Cat# 7076; RRID: AB_330924
Anti-rabbit IgG HRP-linked antibody (1:5000 for WB)	Cell Signaling Technology	Cat# 7074; RRID: AB_2099233
CBP (D6C5, 1:1000 for WB, 1:250 for IP)	Cell Signaling Technology	Cat# 7389; RRID: AB_2616020
CD45 (D3F8Q, 1:1000 for WB)	Cell Signaling Technology	Cat# 70257, RRID: AB_2799780

CREB (48H2, 1:1000 for WB, 1:250 for IP, 1:50 for CUT&RUN)	Cell Signaling Technology	Cat# 9197; RRID: AB_331277
c-Fos (9F6, 1:1000 for WB)	Cell Signaling Technology	Cat# 2250; RRID: AB_2247211
JNK (1:1000 for WB)	Cell Signaling Technology	Cat# 9252; RRID: AB_2250373
K48-linkage specific Polyubiquitin (D9D5, 1:500 for WB)	Cell Signaling Technology	Cat# 8081; RRID: AB_10859893
NF-κB p65 (D14E12, 1:1000 for WB)	Cell Signaling Technology	Cat# 8242; RRID: AB_10859369
p38 MAPK (D13E1, 1:1000 for WB)	Cell Signaling Technology	Cat# 8690; RRID: AB_10999090
p44/42 MAPK (ERK1/2) (137F5, 1:1000 for WB)	Cell Signaling Technology	Cat# 4695; RRID: AB_390779
Phospho-NF-κB p65 (Ser536) (93H1, 1:1000 for WB)	Cell Signaling Technology	Cat# 3033; RRID: AB_331284
Phospho-CREB (Ser133) (87G3, 1:1000 for WB)	Cell Signaling Technology	Cat# 9198; RRID: AB_2561044
Phospho-JNK (Thr183/Tyr185) (81E11, 1:1000 for WB)	Cell Signaling Technology	Cat# 4668; RRID: AB_823588
Phospho-p38 MAPK (Thr180/Tyr182) (D3F9, 1:1000 for WB)	Cell Signaling Technology	Cat# 4511; RRID: AB_2139682
Phospho-p44/42 MAPK (ERK1/2) (Thr202/Tyr204) (D13.14.4E, 1:1000 for WB)	Cell Signaling Technology	Cat# 4370T; RRID: AB_2315112
Phospho-STAT3 (Tyr705) (D3A7, 1:1000 for WB)	Cell Signaling Technology	Cat# 9145; RRID: AB_2491009
RUNX2 (D1L7F, 1:1000 for WB))	Cell Signaling Technology	Cat# 12556; RRID: AB_2732805
STAT3 (D3Z2G, 1:1000 for WB, 1:250 for IP, 1:50 for CUT&RUN)	Cell Signaling Technology	Cat# 12640; RRID: AB_2629499
TLR2 (E1J2W, 1:1000 for WB))	Cell Signaling Technology	Cat# 13744; RRID: AB_2798308
TLR4 (D8L5W, 1:1000 for WB))	Cell Signaling Technology	Cat# 14358; RRID: AB_2798460
P. gingivalis (no clone number, 1:100 for IF)	Creative Diagnostics	Cat# DMAB9447, RRID:AB_2392991
MYD88 (1:100 for IHC)	LSBio	Cat# LS-C357983; RRID: N/A
TLR2 (1:100 for IHC)	Novus Biologicals	Cat# NB100-56720; RRID: AB_838993
PDLIM2 (1:500 for WB)	Origene	Cat# AP32071PU-N
CRTC2 (628430, 1:50 for CUT&RUN)	R&D systems	Cat# MAB6338; RRID: AB_10719421
Goat IgG HRP-conjugated Antibody (1:5000 for WB)	R&D systems	Cat# HAF109; RRID: AB_357236
RANKL (1:500 for WB)	R&D systems	Cat# AF462; RRID: AB_2206198
Sclerostin (1:500 for WB, 1:100 for IHC)	R&D systems	Cat# AF1589; RRID: AB_2195345
TNF-alpha monoclonal antibody (MP6-XT22)	R&D systems	Cat# MAB4101, RRID:AB_2240643
β-Actin (C4, 1:3000 for WB)	Santa Cruz Biotechnology	Cat# sc-47778; Lot#1717; RRID: AB_2714189
c-MYC (9E10, 1:1000 for WB)	Santa Cruz Biotechnology	Cat# sc-40; RRID: AB_10637885
Ly-6G (RB6-8C5, 1:100 for IHC)	Santa Cruz Biotechnology	Cat# sc-53515, RRID:AB_783639
TRAF6 (H-274, 1:1000 for WB)	Santa Cruz Biotechnology	Cat# sc-2771, RRID:AB_1128423
FLAG (M2, 1:1000 for WB)	Sigma-Aldrich	Cat# F1804; RRID: AB_262044
Donkey anti-Goat IgG (H+L) Cross-Adsorbed Secondary Antibody, Alexa Fluor™ 594 (1:500 for IF)	Thermo Fisher Scientific	Cat# A-11058, RRID:AB_142540
Goat anti-Mouse IgG (H+L) Highly Cross-Adsorbed Secondary Antibody, Alexa Fluor™ Plus 488 (1:500 for IF)	Thermo Fisher Scientific	Cat# A32723, RRID:AB_2633275
Goat anti-Rabbit IgG (H+L) Cross-Adsorbed Secondary Antibody, Alexa Fluor™ 594 (1:500 for IF)	Thermo Fisher Scientific	Cat# A-11012, RRID:AB_141359
IL-6 monoclonal antibody (MP5-20F3)	Thermo Fisher Scientific	Cat# 16-7061-38, RRID:AB_2573106
**siRNA**		
c-Fos siRNA (m)	Santa Cruz Biotechnology	Cat# sc-29222
CREB-1 siRNA (m)	Santa Cruz Biotechnology	Cat# sc-35111
IKKα siRNA (m)	Santa Cruz Biotechnology	Cat# sc-29366
IKKβ siRNA (m)	Santa Cruz Biotechnology	Cat# sc-35645
JNK1 siRNA (m)	Santa Cruz Biotechnology	Cat# sc-29381
JNK2 siRNA (m)	Santa Cruz Biotechnology	Cat# sc-39102
MEK1 siRNA (m)	Santa Cruz Biotechnology	Cat# sc-35904

MEK2 siRNA (m)	Santa Cruz Biotechnology	Cat# sc-35906
p38α MAPK14 siRNA (m)	Santa Cruz Biotechnology	Cat# sc-29434
p38β MAPK11 siRNA (m)	Santa Cruz Biotechnology	Cat# sc-39117
p38γ MAPK12 siRNA (m)	Santa Cruz Biotechnology	Cat# sc-39014
RUNX2 siRNA (m)	Santa Cruz Biotechnology	Cat# sc-37146
STAT3 siRNA (m)	Santa Cruz Biotechnology	Cat# sc-29494
STAT5 siRNA (m)	Santa Cruz Biotechnology	Cat# sc-29496
Control siRNA-A	Santa Cruz Biotechnology	Cat# sc-37007
Control siRNA (Fluorescein Conjugate)-A	Santa Cruz Biotechnology	Cat# sc-36869
**Bacteria**		
Porphyromonas gingivalis	ATCC	ATCC 33277
**Lentivirus**		
FBXL19 ORF cDNA lentiviral particles (C-Flag-SV40-mCherry-IRES-puromycin)	GeneCopoeia	LPP-Mm15723-Lv216
Negative control for LPP-Mm15723-Lv216	GeneCopoeia	LPP-NEG-Lv216
PDLIM2 ORF cDNA lentiviral particles (C-Myc-SV40-mCherry-IRES-puromycin)	GeneCopoeia	LPP-Mm14320-Lv204
Negative control for LPP-Mm14320-Lv204	GeneCopoeia	LPP-NEG-Lv204
shRNAs against mouse Fbxl19 (NM_172748.2) in psi-LVE002 with CMV promoter, mCherry, puromycin	GeneCopoeia	LPP-MSE040575-LVE002-100
shRNAs against mouse Pdlim2 (NM_145978.2) in psi-LVE002 with CMV promoter, mCherry, puromycin (miRE-based design)	GeneCopoeia	LPP-MSE095597-LVE002-100
Scrambled control shRNA in psi-LVE002 with CMV promoter, mCherry, puromycin (miRE-based design)	GeneCopoeia	LPP-CSECTR001-LVE002-100
**Chemicals, peptides, and recombinant proteins**		
Hemin (porcine)	Alfa Aesar	Cat# A11165
T6167923	Aobious	Cat# AOB5875
Cycloheximide	APExBIO	Cat# A8244
Leupeptin, Microbial	APExBIO	Cat# A2570
Sulfamethoxazole/trimethoprim suspension	Aurobindo Pharma USA	Cat# 65862-496-47
Tryptic Soy Broth	BD Bacto™	Cat# DF0370-17-3
Yeast Extract	BD Bacto™	Cat# 212750
Rat tail type I collagen coating solution	Cell Applications	Cat# 122-20
SB203580	Cell Signaling Technology	Cat# 5633
SP600125	Cell Signaling Technology	Cat# 8177
U0126	Cell Signaling Technology	Cat# 9903
Dimethyl Sulfoxide (DMSO)	Fisher Bioreagents	Cat# BP2311
Penicillin/Streptomycin	Fisher Scientific	Cat# SV30010
Proteinase K, RNase/DNase free	Gold Biotechnology	Cat# P-480-100
FLA-ST	Invivogen	Cat# tlrl-stfla
LPS from *E. coli*	Invivogen	Cat# tlrl-3pelps
LPS from *P. gingivalis*	Invivogen	Cat# tlrl-pglps
LTA from *S. aureus*	Invivogen	Cat# tlrl-pslta
Pam2CSK4	Invivogen	Cat# tlrl-pm2s-1
Pam3CSK4	Invivogen	Cat# tlrl-pms
Single-strands RNA	Invivogen	N/A
CADD522	MedChemExpress	Cat# HY-107999
C188-9	MedChemExpress	Cat# HY-112288
H-89	MedChemExpress	Cat# HY-15979A
STAT5-IN-1	MedChemExpress	Cat# HY-101853
T-5224	MedChemExpress	Cat# HY-12270

666-15	MedChemExpress	Cat# HY-101120
Carboxymethylcellulose, sodium salt, high viscosity	Millipore	Cat# 217274
TransIT-X2® dynamic delivery system	Mirus	Cat# MIR 6000
Menadione	MP Biomedicals	Cat# 102259
2-Mercaptoethanol	MP Biomedicals	Cat# 194705
Recombinant Murine M-CSF	PeproTech	Cat# 315-02
Normal rat IgG	Santa Cruz Biotechnology	Cat# sc-2026, RRID:AB_737202
Normal rabbit IgG	Santa Cruz Biotechnology	Cat# sc-2027, RRID:AB_737197
Normal goat IgG	Santa Cruz Biotechnology	Cat# sc-2028, RRID:AB_737167
Protein A/G PLUS-Agarose	Santa Cruz Biotechnology	Cat# sc-2003, RRID:AB_10201400
MG132	Selleckchem	Cat# A11043
BMS-345541	Sigma-Aldrich	Cat# B9935
Donkey serum	Sigma-Aldrich	Cat# D9663, RRID:AB_2810235
Goat serum	Sigma-Aldrich	Cat# G9023
Hexadimethrine bromide (Polybrene)	Sigma-Aldrich	Cat# 28728-55-4
Phosphatase Inhibitor Cocktail 2	Sigma-Aldrich	Cat# P5726
Phosphatase Inhibitor Cocktail 3	Sigma-Aldrich	Cat# P0044
Protease inhibitor cocktail	Sigma-Aldrich	Cat# P8340
Alexa Fluor™ 488 Phalloidin	Thermo Fisher Scientific	Cat# A12379
AnaeroPack™-Anaero Anaerobic Gas Generator	Thermo Fisher Scientific	Cat# 23-246-376
DAPI	Thermo Fisher Scientific	Cat# D1306, RRID:AB_2629482
PowerUp™ SYBR™ Green master mix	Thermo Fisher Scientific	Cat# A25743
Collagenase, Type 1, Filtered	Worthington Biochemical Corporation	LS004217
RiboZol™ RNA Extraction Reagent	VWR Life Science	Cat# N580
**Assay kit**		
Murine sRANK Ligand pre-coated ELISA kit	biogems	Cat# BGK35235
MojoSort™ mouse neutrophil isolation kit	BioLegend	Cat# 480017
CUT & RUN assay kit	Cell Signaling Technology	Cat# 86652
MagniSort™ mouse CD45 depletion kit	Invitrogen	Cat# 8804-6864-74
High-capacity cDNA reverse transcription kit	Life Technologies	Cat# 4368814
DNeasy blood & tissue kit	Qiagen	Cat# 69504
Acid phosphatase, leukocyte (TRAP) kit	Sigma-Aldrich	Cat# 387A
Pierce™ BCA protein assay kit	Thermo Fisher Scientific	Cat# 23225
M.O.M.® (Mouse on Mouse) immunodetection kit	Vector Laboratories	Cat# BMK-2202
**Cell line**		
IDG-SW3	Provided by Lynda Bonewald	This paper
MLO-Y4	Provided by Lynda Bonewald	This paper
**Mice**		
C57BL/6J	Jackson Laboratory	JAX:000664
B6N.FVB-Tg(Dmp1-cre)1Jqfe/BwdJ	Jackson Laboratory	JAX:023047
B6.FVB-Tg(BGLAP-cre)1Clem/J	Jackson Laboratory	JAX:019509
B6.129P2(SJL)-*Myd88*^{tm1Defr}/J	Jackson Laboratory	JAX:008888
B6.129-*Tnfsf11*$^{tm1.1Caob}$/J	Jackson Laboratory	JAX:018978
B6.129(Cg)-*Il1r1*$^{tm1.1Rbl}$/J	Jackson Laboratory	JAX:028398
B6.129-*Tlr2tm1Kir/*J	Jackson Laboratory	JAX:004650
B6. B10ScN-*Tlr4*$^{lps\text{-}del}$/JthJ	Jackson Laboratory	JAX:007227
B6.129P2-*Myd88*^{tm1Hlz}/J	Jackson Laboratory	JAX:028939
B6.129P2(SJL)-*Myd88*$^{tm1.1Defr}$/J	Jackson Laboratory	JAX:009088

B6.129S7-*Rag1*^{tm1Mom}/J	Jackson Laboratory	JAX:002216
Tlr2$^{fl/fl}$ mice on the C57BL/6 background	Provided by W. Henry Boom (doi: https://doi.org/10.1101/2021.05.19.444905)	This paper
**Oligonucleotide**		
Primers for RT-qPCR	This paper	Supplementary Tables 1
Primers for CUT & RUN-qPCR	This paper	Supplementary Tables 3
Primers for genomic DNA PCR	This paper	Supplementary Tables 4
**Software and algorithm**		
QuantStudio Real-Time PCR systems	Thermo Fisher Scientific	https://www.thermofisher.com/us/en/home/life-science/pcr/real-time-pcr/real-time-pcr-instruments/quantstudio-systems.html
Graphpad Prism v9.4.1	GraphPad Software	N/A
Bioquant Osteo	Bioquant Image Analysis Corporation	https://bioquant.com/
NRecon	Bruker	https://www.bruker.com/en/products-and-solutions/microscopes/3d-x-ray-microscopes.html
DATA viewer, CTVox software, CT Analyser	Bruker	https://www.bruker.com/en/products-and-solutions/microscopes/3d-x-ray-microscopes/xrm-software.html
ImageJ (FIJI, enhanced version of ImageJ2)	Schneider et al., 2012	https://imagej.nih.gov/ij/

Supplementary Table 3: Primers for CUT & RUN assays.

	Forward (5'-3')	Reverse (5'-3')
Rankl TSS (promoter) [1]	cagaaaccaaccactggacccaa	caggaacatggagcgggagg
Rankl D1 [1]	gagaaagccaagtcctgggt	cattctggggccggaacaaa
Rankl D2 [2]	cttggaaggactccaggaaa	cctttctcagagcacactgg
Rankl D3 [2]	aaatcccatttgctttccag	gagctgtgtcctagaagaattgtc
Rankl D4 [2]	tgggagactcagttgttgct	tgttgttggttcgttgtcct
Rankl D5 [2]	gatggagtcaggatgcacag	gagccctgagaacagtgtga
Rankl D6 [2]	gaagagaacattgctggttgc	taaggatgctttcccagctc
Rankl D7 [2]	cacctgtaattctagcacgca	tcacgctcctctcaaattca
Rankl T1 [2]	tggtccaggtcaagcaataa	ggcaacacaaacctcctgta
Rankl T2 [2]	cctctgggagcaaatgagag	ggtgcatctgtggatggtaa
Rankl T3 [2]	ccttgaattctttggactgga	tacactgtcctttccttgcg

[1] Kim, S. *et al. Mol Endocrinol* 2007, 21 (1), 197-214
[2] Wein, MN. *et al. Nat Commun* 2016, 7:13176

Supplementary Table 4: Primers for detecting *Myd88* gene recombination.

	5'-3'
Myd88-delta-F2	aggtagaataggtgggccac
Myd88-fl-R1	gtcagaaacaaccaccaccatgc
Myd88-LSL-common	accagaaagggcgtcaga
Myd88-LSL-R4	atgccccaggttgctgctgtac

1 Kim, S., Yamazaki, M., Shevde, N. K. & Pike, J. W. Transcriptional control of receptor activator of nuclear factor-kappaB ligand by the protein kinase A activator forskolin and the transmembrane glycoprotein 130-activating cytokine, oncostatin M, is exerted through multiple distal enhancers. *Mol Endocrinol* **21**, 197-214 (2007).
2 Wein, M. N. *et al.* SIKs control osteocyte responses to parathyroid hormone. *Nat Commun* **7**, 13176 (2016).
