# Supplementary material for: Osteocytes directly regulate osteolysis via MYD88 signaling in bacterial bone infection
Source: Nat Commun. 2022 Nov 4;13:6648. doi: 10.1038/s41467-022-34352-z (PMC9636212; doi:10.1038/s41467-022-34352-z)
Supplement: Supplementary file 3 — Reporting Summary [file 41467_2022_34352_MOESM3_ESM.pdf]

nature portfolio

Corresponding author(s): Yasuyoshi Ueki

Last updated by author(s): Oct 10, 2022

Reporting Summary

Nature Portfolio wishes to improve the reproducibility of the work that we publish. This form provides structure for consistency and transparency in reporting. For further information on Nature Portfolio policies, see our Editorial Policies and the Editorial Policy Checklist.

Statistics

For all statistical analyses, confirm that the following items are present in the figure legend, table legend, main text, or Methods section.

n/a	Confirmed	
☐	☒	The exact sample size (*n*) for each experimental group/condition, given as a discrete number and unit of measurement
☐	☒	A statement on whether measurements were taken from distinct samples or whether the same sample was measured repeatedly
☐	☒	The statistical test(s) used AND whether they are one- or two-sided<br>*Only common tests should be described solely by name; describe more complex techniques in the Methods section.*
☒	☐	A description of all covariates tested
☐	☒	A description of any assumptions or corrections, such as tests of normality and adjustment for multiple comparisons
☐	☒	A full description of the statistical parameters including central tendency (e.g. means) or other basic estimates (e.g. regression coefficient) AND variation (e.g. standard deviation) or associated estimates of uncertainty (e.g. confidence intervals)
☐	☒	For null hypothesis testing, the test statistic (e.g. *F*, *t*, *r*) with confidence intervals, effect sizes, degrees of freedom and *P* value noted<br>*Give P values as exact values whenever suitable.*
☒	☐	For Bayesian analysis, information on the choice of priors and Markov chain Monte Carlo settings
☒	☐	For hierarchical and complex designs, identification of the appropriate level for tests and full reporting of outcomes
☒	☐	Estimates of effect sizes (e.g. Cohen's *d*, Pearson's *r*), indicating how they were calculated

Our web collection on statistics for biologists contains articles on many of the points above.

Software and code

Policy information about availability of computer code

Data collection	• Data for qPCR were acquired with the QuantStudio design & analysis software (Thermo Fisher).<br>• Mice or tissues were scanned with the Skyscan1176 (Bruker).<br>• Bands for Western blotting were visualized with the Celvin S 320+ Chemiluminescence Imager (Biostep).<br>• Data for ELISA were acquired with the SpectraMax® i3 (Molecular Devices).<br>• Images of TRAP staining, H&E staining, Immunohistochemical staining, and immunofluorescent staining were acquired with the BZ-X800 microscope (Keyence).
Data analysis	Statistical analyses were performed with Prism 9.4.1 (GraphPad). ImageJ (FIJI, enhanced version of ImageJ2, https://imagej.nih.gov/ij/). qPCR data were analyzed with the QuantStudio design & analysis software (Thermo Fisher). Data scanned with Skyscan1176 were reconstructed with NRecon software (Bruker). 3D Images were aligned with DataViewer (Bruker), created with CTVox software (Bruker), and analyzed with CTAnalyser (Bruker).<br>The number of osteoclasts/bone surface and osteoclast surface/bone surface were measured by Bioquant Osteo software in a blinded manner. Fluorescence intensities were acquired with SpectraMax® i3 and were analyzed with SoftMax® Pro6 (Molecular Devices).

For manuscripts utilizing custom algorithms or software that are central to the research but not yet described in published literature, software must be made available to editors and reviewers. We strongly encourage code deposition in a community repository (e.g. GitHub). See the Nature Portfolio guidelines for submitting code & software for further information.

Data

Policy information about availability of data

All manuscripts must include a data availability statement. This statement should provide the following information, where applicable:

- Accession codes, unique identifiers, or web links for publicly available datasets
- A description of any restrictions on data availability
- For clinical datasets or third party data, please ensure that the statement adheres to our policy

All data supporting the findings of this study are available within this article and its supplementary information files. Source data are provided with this paper.

Human research participants

Policy information about studies involving human research participants and Sex and Gender in Research.

Reporting on sex and gender	Not applicable
Population characteristics	Not applicable
Recruitment	Not applicable
Ethics oversight	Not applicable

Note that full information on the approval of the study protocol must also be provided in the manuscript.

Field-specific reporting

Please select the one below that is the best fit for your research. If you are not sure, read the appropriate sections before making your selection.

☒ Life sciences ☐ Behavioural & social sciences ☐ Ecological, evolutionary & environmental sciences

For a reference copy of the document with all sections, see nature.com/documents/nr-reporting-summary-flat.pdf

Life sciences study design

All studies must disclose on these points even when the disclosure is negative.

Sample size	The number of replicates is mentioned in the figure legends and the corresponding data point for each independent replicate are shown in the graphs for all experiments. No statistical test was used to pre-determine sample size. However, the sample sizes used for the experiments in the manuscript were based on our previous experience. For example, in vivo experiments to compare mice strains or conditions consist of more than three replicates, as in previous studies we published (Kittaka et al. J Bone Miner Res. 2020. DOI: 10.1002/jbmr.3882; Kittaka et al. Bone Rep. 2020. DOI: 10.1016/j.bonr.2020.100258; Yoshimoto et al. J Bone Miner Res. 2018. DOI: 10.1002/jbmr.3449). Similarly, in vitro experiments consisted of more than three independent experiments to compare cellular responses based on our previous studies (Kittaka et al. J Bone Miner Res. 2020. DOI: 10.1002/jbmr.3882; Kittaka et al. J Bone Miner Res. 2018. doi: 10.1002/jbmr.3295).
Data exclusions	No samples or animals were excluded from the analyses.
Replication	The experimental findings were reproduced in multiple independent experiments. The number of independent experiments and biological replicates for each data panel was described in corresponding figure legends. Data in a minority of panels are a representative experiment (e.g. for immunofluorescence images or for Western blotting) and in those cases the number of independent experiments that reproduced the finding is also indicated in the figure legends.
Randomization	Animals or cells were allocated randomly into the different experimental group.
Blinding	Grouping during data collection in mouse experiments, histomorphometric analysis of bone, micro-CT analysis, and counting of TRAP-positive cells in co-culture experiments were performed and analyzed in a blinded fashion. For experiments with samples derived from in vivo experiments (gene and protein expression), the investigator organizing the experimental groups and involved in sample collection was not blinded, and colleagues aiding in data collection were blinded. For in vitro experiments, the investigators were not blinded for group allocation as the same investigator both planned and performed the experiment.

Reporting for specific materials, systems and methods

We require information from authors about some types of materials, experimental systems and methods used in many studies. Here, indicate whether each material, system or method listed is relevant to your study. If you are not sure if a list item applies to your research, read the appropriate section before selecting a response.

Materials & experimental systems

n/a	Involved in the study
☐	☒ Antibodies
☐	☒ Eukaryotic cell lines
☒	☐ Palaeontology and archaeology
☐	☒ Animals and other organisms
☒	☐ Clinical data
☒	☐ Dual use research of concern

Methods

n/a	Involved in the study
☒	☐ ChIP-seq
☒	☐ Flow cytometry
☒	☐ MRI-based neuroimaging

Antibodies

Antibodies used

The following antibodies were used.
Abcam:
• Recombinant Anti-FBXL19 antibody [EPR11957] (Cat# ab172961; RRID: AB_2801257, 1:1000 for Western blotting)
• Anti-Keratocan/KTN antibody (Cat# ab128304; RRID: AB_11144483, 1:500 for Western blotting, 1:100 for Immunofluorescent staining)
• Anti-MyD88 antibody (Cat# ab135693; RRID: AB_2802168, 1:1000 for Western blotting)
• Anti-TLR4 antibody (Cat# ab13867; RID: AB_300696, 1:100 for Immunohistochemical staining)

Bio-Rad:
• F4/80 Antibody|Cl:A3-1 (Cat# MCA497R; RRID:AB_323279, 1:200 for Immunohistochemical staining)

Cell Signaling Technology:
• Anti-mouse IgG, HRP-linked Antibody (Cat# 7076; RRID: AB_330924, 1:5000 for Western blotting)
• Anti-rabbit IgG, HRP-linked Antibody (Cat# 7074; RRID: AB_2099233, 1:5000 for Western blotting)
• CBP (D6C5) Rabbit mAb (Cat# 7389; RRID: AB_2616020, 1:1000 for Western blotting, 1:250 for Immunoprecipitation)
• CD45 (D3F8Q) Rabbit mAb (Cat# 70257, RRID: AB_2799780, 1:1000 for Western blotting)
• CREB (48H2) Rabbit mAb (Cat# 9197; RRID: AB_331277, 1:1000 for Western blotting, 1:250 for Immunoprecipitation, 1:50 for CUT&RUN)
• c-Fos (9F6) Rabbit mAb (Cat# 2250; RRID: AB_2247211, 1:1000 for Western blotting)
•SAPK/JNK Antibody (Cat# 9252; RRID: AB_2250373, 1:1000 for Western blotting)
• K48-linkage Specific Polyubiquitin (D9D5) Rabbit mAb (Cat# 8081; RRID: AB_10859893, 1:500 for Western blotting)
• NF-κB p65 (D14E12) XP® Rabbit mAb (Cat# 8242; RRID: AB_10859369, 1:1000 for Western blotting)
• p38 MAPK (D13E1) XP® Rabbit mAb (Cat# 8690; RRID: AB_10999090, 1:1000 for Western blotting)
• p44/42 MAPK (Erk1/2) (137F5) Rabbit mAb (Cat# 4695; RRID: AB_390779, 1:1000 for Western blotting)
• Phospho-NF-κB p65 (Ser536) (93H1) Rabbit mAb (Cat# 3033; RRID: AB_331284, 1:1000 for Western blotting)
• Phospho-CREB (Ser133) (87G3) Rabbit mAb (Cat# 9198; RRID: AB_2561044, 1:1000 for Western blotting)
• Phospho-SAPK/JNK (Thr183/Tyr185) (81E11) Rabbit mAb (Cat# 4668; RRID: AB_823588, 1:1000 for Western blotting)
• Phospho-p38 MAPK (Thr180/Tyr182) (D3F9) XP® Rabbit mAb (Cat# 4511; RRID: AB_2139682, 1:1000 for Western blotting)
• Phospho-p44/42 MAPK (Erk1/2) (Thr202/Tyr204) (D13.14.4E) XP® Rabbit mAb (Cat# 4370; RRID: AB_2315112, 1:1000 for Western blotting)
• Phospho-Stat3 (Tyr705) (D3A7) XP® Rabbit mAb (Cat# 9145; RRID: AB_2491009, 1:1000 for Western blotting)
• RUNX2 (D1L7F) Rabbit mAb (Cat# 12556; RRID: AB_2732805, 1:1000 for Western blotting)
• Stat3 (D3Z2G) Rabbit mAb (Cat# 12640; RRID: AB_2629499, 1:1000 for Western blotting, 1:250 for Immunoprecipitation, 1:50 for CUT&RUN)
• Toll-like Receptor 2 (E1J2W) Rabbit mAb (Mouse Specific) (Cat# 13744; RRID: AB_2798308, 1:1000 for Western blotting)
• Toll-like Receptor 4 (D8L5W) Rabbit mAb (Mouse Specific) (Cat# 14358; RRID: AB_2798460, 1:1000 for Western blotting)

Creative Diagnostics:
• Anti-P. gingivalis Monoclonal antibody (No clone number. Cat# DMAB9447, RRID:AB_2392991, 1:100 for Immunofluorescent staining)

Lsbio:
• Polyclonal Rabbit anti-Human MYD88 Antibody (IHC, WB) (Cat# LS-C357983; RRID: N/A, 1:100 for Immunohistochemical staining)

Novus Biologicals:
• TLR2 Antibody (Cat# NB100-56720; RRID: AB_838993, 1:100 for Immunohistochemical staining)

Origene:
• PDLIM2 Goat Polyclonal Antibody (Cat# AP32071PU-N; RRID: N/A, 1:500 for Western blotting)

R&D systems:
• Human/Mouse TORC2 Antibody (Cat# MAB6338; RRID: AB_10719421, 1:50 for CUT&RUN)
• Goat IgG HRP-conjugated Antibody (Cat# HAF109; RRID: AB_357236, 1:5000 for Western blotting)
• Mouse TRANCE/TNFSF11/RANK L Antibody (Cat# AF462; RRID: AB_2206198, 1:500 for Western blotting)
• Mouse SOST/Sclerostin Antibody (Cat# AF1589; RRID: AB_2195345, 1:500 for Western blotting, 1:100 for Immunofluorescent staining)

• Mouse TNF-alpha Antibody (MP6-XT22) (Cat# MAB4101, RRID:AB_2240643)

Santa Cruz Biotechnology:
• Anti-β-Actin Antibody (C4) (Cat# sc-47778; Lot#1717; RRID: AB_2714189, 1:3000 for Western blotting)
• Anti-Myc/c-Myc Antibody (9E10) (Cat# sc-40; RRID: AB_10637885, 1:1000 for Western blotting)
• Anti-Ly-6G Antibody (RB6-8C5) (Cat# sc-53515, RRID:AB_783639, 1:100 for Immunohistochemical staining)
• TRAF6 Antibody (H-274) (Cat# sc-2771, RRID:AB_1128423, 1:1000 for Western blotting)

Sigma-Aldrich:
• Monoclonal ANTI-FLAG® M2 antibody produced in mouse (Cat# F1804; RRID: AB_262044, 1:1000 for Western blotting)
The following antibody (Thermo Fisher Scientific) against
• Donkey anti-Goat IgG (H+L) Cross-Adsorbed Secondary Antibody, Alexa Fluor™ 594 (Cat# A-11058, RRID:AB_142540, 1:500 for Immunofluorescent staining)
• Goat anti-Mouse IgG (H+L) Highly Cross-Adsorbed Secondary Antibody, Alexa Fluor™ Plus 488 (Cat# A32723, RRID:AB_2633275, 1:500 for Immunofluorescent staining)
• Goat anti-Rabbit IgG (H+L) Cross-Adsorbed Secondary Antibody, Alexa Fluor™ 594 (Cat# A-11012, RRID:AB_141359, 1:500 for Immunofluorescent staining)
• IL-6 Monoclonal Antibody (MP5-20F3), Functional Grade, eBioscience™ (Cat# 16-7061-38, RRID:AB_2573106)

Validation

All antibodies used in this study are commercially available and have been validated by the vender's and the previous published studies. The antibodies, except for anti-MYD88 antibody (LSBio, Cat# LS-C357983; RRID: N/A) and anti-PDLIM2 antibody (Origene, Cat# AP32071PU-N; RRID: N/A), have been assigned a Research Resource Identifiers (#RRID) to promote reproducibility in scientific research.

The following antibodies (Abcam) were validated as shown in the vender's website.
• Recombinant Anti-FBXL19 antibody [EPR11957] (Cat# ab172961; RRID: AB_2801257, 1:1000 for Western blotting, https://www.abcam.com/fbxl19-antibody-epr11957-ab172961.html)
• Anti-Keratocan/KTN antibody (Cat# ab128304; RRID: AB_11144483, 1:500 for Western blotting, 1:100 for Immunofluorescent staining, https://www.abcam.com/keratocanktn-antibody-ab128304.html). The antibody for Immunofluorescent was also validated by previous published study (Türker et al. 2018. Front Endocrinol (Lausanne).)
• Anti-MyD88 antibody (Cat# ab135693; RRID: AB_2802168, 1:1000 for Western blotting, https://www.abcam.com/myd88-antibody-ab135693.html). The antibody was also validated by previous published studies (Hamie et al. 2021. Front Immunol., PMID: 33767699; Zhang et al. 2019. Molecules, PMID: 31861525)
• Anti-TLR4 antibody (Cat# ab13867; RID: AB_300696, 1:100 for Immunohistochemical staining, https://www.abcam.com/tlr4-antibody-ab13867.html).

The following antibodies (Bio-Rad) were validated as shown in the vender's website.
• F4/80 Antibody|Cl:A3-1 (Cat# MCA497R; RRID:AB_323279, 1:200 for Immunohistochemical staining, https://www.bio-rad-antibodies.com/monoclonal/mouse-f4-80-antibody-cl-a3-1-mca497.html
f=purified&JSESSIONID_STERLING=0C8730700BAB5153AB4CEE0B0C6A959F.ecommerce1&evCntryLang=US-en&cntry=US&thirdPartyCookieEnabled=true)

The following antibodies (Cell Signaling Technology) were validated as shown in the vender's website.
• Anti-mouse IgG, HRP-linked Antibody (Cat# 7076; RRID: AB_330924, 1:5000 for Western blotting, https://www.cellsignal.com/products/secondary-antibodies/anti-mouse-igg-hrp-linked-antibody/7076)
• Anti-rabbit IgG, HRP-linked Antibody (Cat# 7074; RRID: AB_2099233, 1:5000 for Western blotting, https://www.cellsignal.com/products/secondary-antibodies/anti-rabbit-igg-hrp-linked-antibody/7074)
• CBP (D6C5) Rabbit mAb (Cat# 7389; RRID: AB_2616020, 1:1000 for Western blotting, 1:250 for Immunoprecipitation,https://www.cellsignal.com/products/primary-antibodies/cbp-d6c5-rabbit-mab/7389)
• CD45 (D3F8Q) Rabbit mAb (Cat# 70257, RRID: AB_2799780, 1:1000 for Western blotting, https://www.cellsignal.com/products/primary-antibodies/cd45-d3f8q-rabbit-mab/70257)
• CREB (48H2) Rabbit mAb (Cat# 9197; RRID: AB_331277, 1:1000 for Western blotting, 1:250 for Immunoprecipitation, 1:50 for CUT&RUN, https://www.cellsignal.com/products/primary-antibodies/creb-48h2-rabbit-mab/9197)
• c-Fos (9F6) Rabbit mAb (at# 2250; RRID: AB_2247211, 1:1000 for Western blotting, https://www.cellsignal.com/products/primary-antibodies/c-fos-9f6-rabbit-mab/2250)
• SAPK/JNK Antibody (Cat# 9252; RRID: AB_2250373, 1:1000 for Western blotting, https://www.cellsignal.com/products/primary-antibodies/sapk-jnk-antibody/9252)
• K48-linkage Specific Polyubiquitin (D9D5) Rabbit mAb (Cat# 8081; RRID: AB_10859893, 1:500 for Western blotting, https://www.cellsignal.com/products/primary-antibodies/k48-linkage-specific-polyubiquitin-d9d5-rabbit-mab/8081)
• NF-κB p65 (D14E12) XP® Rabbit mAb (Cat# 8242; RRID: AB_10859369, 1:1000 for Western blotting, https://www.cellsignal.com/products/primary-antibodies/nf-kb-p65-d14e12-xp-rabbit-mab/8242)
• p38 MAPK (D13E1) XP® Rabbit mAb (Cat# 8690; RRID: AB_10999090, 1:1000 for Western blotting, https://www.cellsignal.com/products/primary-antibodies/p38-mapk-d13e1-xp-rabbit-mab/8690)
• p44/42 MAPK (Erk1/2) (137F5) Rabbit mAb (Cat# 4695; RRID: AB_390779, 1:1000 for Western blotting, https://www.cellsignal.com/products/primary-antibodies/p44-42-mapk-erk1-2-137f5-rabbit-mab/4695)
• Phospho-NF-κB p65 (Ser536) (93H1) Rabbit mAb (Cat# 3033; RRID: AB_331284, 1:1000 for Western blotting, https://www.cellsignal.com/products/primary-antibodies/phospho-nf-kb-p65-ser536-93h1-rabbit-mab/3033)
• Phospho-CREB (Ser133) (87G3) Rabbit mAb (Cat# 9198; RRID: AB_2561044, 1:1000 for Western blotting, https://www.cellsignal.com/products/primary-antibodies/phospho-creb-ser133-87g3-rabbit-mab/9198)
• Phospho-SAPK/JNK (Thr183/Tyr185) (81E11) Rabbit mAb (Cat# 4668; RRID: AB_823588, 1:1000 for Western blotting, https://www.cellsignal.com/products/primary-antibodies/phospho-sapk-jnk-thr183-tyr185-81e11-rabbit-mab/4668)
• Phospho-p38 MAPK (Thr180/Tyr182) (D3F9) XP® Rabbit mAb (Cat# 4511; RRID: AB_2139682, 1:1000 for Western blotting, https://www.cellsignal.com/products/primary-antibodies/phospho-p38-mapk-thr180-tyr182-d3f9-xp-rabbit-mab/4511)
• Phospho-p44/42 MAPK (Erk1/2) (Thr202/Tyr204) (D13.14.4E) XP® Rabbit mAb (Cat# 4370; RRID: AB_2315112, 1:1000 for Western blotting, https://www.cellsignal.com/products/primary-antibodies/phospho-p44-42-mapk-erk1-2-thr202-tyr204-d13-14-4e-xp-

rabbit-mab/4370?site-search-type=Products&N=4294956287&Ntt=4370t&fromPage=plp&_requestid=8382184)
• Phospho-Stat3 (Tyr705) (D3A7) XP® Rabbit mAb (Cat# 9145; RRID: AB_2491009, 1:1000 for Western blotting, https://www.cellsignal.com/products/primary-antibodies/phospho-stat3-tyr705-d3a7-xp-rabbit-mab/9145)
• RUNX2 (D1L7F) Rabbit mAb (Cat# 12556; RRID: AB_2732805, 1:1000 for Western blotting, https://www.cellsignal.com/products/primary-antibodies/runx2-d1l7f-rabbit-mab/12556)
• Stat3 (D3Z2G) Rabbit mAb (Cat# 12640; RRID: AB_2629499, 1:1000 for Western blotting, 1:250 for Immunoprecipitation, 1:50 for CUT&RUN, https://www.cellsignal.com/products/primary-antibodies/stat3-d3z2g-rabbit-mab/12640)
• Toll-like Receptor 2 (E1J2W) Rabbit mAb (Mouse Specific) (Cat# 13744; RRID: AB_2798308, 1:1000 for Western blotting, https://www.cellsignal.com/products/primary-antibodies/toll-like-receptor-2-e1j2w-rabbit-mab-mouse-specific/13744)
• Toll-like Receptor 4 (D8L5W) Rabbit mAb (Mouse Specific) (Cat# 14358; RRID: AB_2798460, 1:1000 for Western blotting, https://www.cellsignal.com/products/primary-antibodies/toll-like-receptor-4-d8l5w-rabbit-mab-mouse-specific/14358)

The following antibody (Creative Diagnostics) were validated as shown in the vender's website.
• Anti-P. gingivalis Monoclonal antibody (Cat# DMAB9447, RRID:AB_2392991, 1:100 for Immunofluorescent staining, https://www.creative-diagnostics.com/Anti-Bacteria-Porphyromonas-Gingivalis-Antibody-53269-144.htm). The antibody for Immunofluorescent staining was also validated by previous published study (Liang et al. 2018. Mol Oral Microbiol. PMID: 29754448)

The following antibody (Lsbio) were validated as shown in the vender's website.
• Polyclonal Rabbit anti-Human MYD88 Antibody (IHC, WB) (Cat# LS-C357983; RRID: N/A, 1:100 for Immunohistochemical staining, https://www.lsbio.com/antibodies/myd88-antibody-ihc-wb-western-ls-c357983/369528)

The following antibody (Novus Biologicals) were validated as shown in the vender's website.
• TLR2 Antibody (Cat# NB100-56720; RRID: AB_838993, 1:100 for Immunohistochemical staining, https://www.novusbio.com/products/tlr2-antibody_nb100-56720)

The following antibody (Origene) were validated as shown in the vender's website.
• PDLIM2 Goat Polyclonal Antibody (Cat# AP32071PU-N; RRID: N/A, 1:500 for Western blotting, https://www.origene.com/catalog/antibodies/primary-antibodies/ap32071pu-n/pdlim2-goat-polyclonal-antibody)

The following antibodies (R&D systems) were validated as shown in the vender's website.
• Human/Mouse TORC2 Antibody (Cat# MAB6338; RRID: AB_10719421, 1:50 for CUT&RUN, https://www.rndsystems.com/products/human-mouse-torc2-antibody-628430_mab6338) .The antibody was also validated by previous published study (Wein et al. 2016. Nat Commun. PMID: 27759007).
• Goat IgG HRP-conjugated Antibody (Cat# HAF109; RRID: AB_357236, 1:5000 for Western blotting, https://www.rndsystems.com/products/goat-igg-hrp-conjugated-antibody_haf109)
• Mouse TRANCE/TNFSF11/RANK L Antibody (Cat# AF462; RRID: AB_2206198, 1:500 for Western blotting, https://www.rndsystems.com/products/mouse-trance-tnfsf11-rank-l-antibody_af462)
• Mouse SOST/Sclerostin Antibody (Cat# AF1589; RRID: AB_2195345, 1:500 for Western blotting, 1:100 for Immunofluorescent staining, https://www.rndsystems.com/products/mouse-sost-sclerostin-antibody_af1589). The antibody for Immunofluorescent staining was also validated by previous published study (Leupin et al. 2007. J Bone Miner Res. PMID: 17696759)
• Mouse TNF-alpha Antibody (MP6-XT22) (Cat# MAB4101, RRID:AB_2240643, https://www.rndsystems.com/products/mouse-tnf-alpha-antibody-mp6-xt22_mab4101)

The following antibodies (Santa Cruz Biotechnology) were validated as shown in the vender's website.
• Anti-β-Actin Antibody (C4) (Cat# sc-47778; Lot#1717; RRID: AB_2714189, 1:3000 for Western blotting, https://www.scbt.com/p/beta-actin-antibody-c4?requestFrom=search)
• Anti-Myc/c-Myc Antibody (9E10) (Cat# sc-40; RRID: AB_10637885, 1:1000 for Western blotting, https://www.scbt.com/ja/p/c-myc-antibody-9e10?requestFrom=search)
• Anti-Ly-6G Antibody (RB6-8C5) (Cat# sc-53515, RRID:AB_783639, 1:100 for Immunohistochemical staining, https://www.scbt.com/p/ly-6g-antibody-rb6-8c5?requestFrom=search). The same lot was also validated by other supplier (https://www.novusbio.com/products/ly-6g-ly-6c-antibody-rb6-8c5_nbp2-00441)
• TRAF6 Antibody (H-274) (Cat# sc-2771, RRID:AB_1128423, 1:1000 for Western blotting, https://www.scbt.com/p/traf6-antibody-h-274. This antibody has been discontinued and replaced by TRAF6 (D-10): sc-8409.

The following antibody (Sigma-Aldrich) against
• Monoclonal ANTI-FLAG® M2 antibody produced in mouse (Cat# F1804; RRID: AB_262044, 1:1000 for Western blotting, https://www.sigmaaldrich.com/US/en/product/sigma/f1804)

The following antibodies (Thermo Fisher Scientific) were validated as shown in the vender's website.
• Donkey anti-Goat IgG (H+L) Cross-Adsorbed Secondary Antibody, Alexa Fluor™ 594 (Cat# A-11058, RRID:AB_142540, 1:500 for Immunofluorescent staining, https://www.thermofisher.com/antibody/product/Donkey-anti-Goat-IgG-H-L-Cross-Adsorbed-Secondary-Antibody-Polyclonal/A-11058)
• Goat anti-Mouse IgG (H+L) Highly Cross-Adsorbed Secondary Antibody, Alexa Fluor™ Plus 488 (Cat# A32723, RRID:AB_2633275, 1:500 for Immunofluorescent staining, https://www.thermofisher.com/antibody/product/Goat-anti-Mouse-IgG-H-L-Highly-Cross-Adsorbed-Secondary-Antibody-Polyclonal/A32723)
• Goat anti-Rabbit IgG (H+L) Cross-Adsorbed Secondary Antibody, Alexa Fluor™ 594 (Cat# A-11012, RRID:AB_141359, 1:500 for Immunofluorescent staining, https://www.thermofisher.com/antibody/product/Goat-anti-Rabbit-IgG-H-L-Cross-Adsorbed-Secondary-Antibody-Polyclonal/A-11012)
• IL-6 Monoclonal Antibody (MP5-20F3), Functional Grade, eBioscience™ (Cat# 16-7061-38, RRID:AB_2573106, https://www.thermofisher.com/antibody/product/IL-6-Antibody-clone-MP5-20F3-Monoclonal/16-7061-38)

Eukaryotic cell lines

Policy information about cell lines and Sex and Gender in Research

Cell line source(s)	IDG-SW3 cells and MLOY-4 cells were provided by Dr. Lynda F. Bonewald, who established these cell lines.
Authentication	IDG-SW3 and MLOY-4 cells were authenticated based on our vast experience working with these cell lines (such as cell morphology and culture condition etc.).
Mycoplasma contamination	Cells were routinely tested for mycoplasma contamination, only negative cells were used in experiments.
Commonly misidentified lines (See ICLAC register)	None of the cell lines used are listed in the database of commonly misidentified cell lines maintained by ICLAC

Animals and other research organisms

Policy information about studies involving animals; ARRIVE guidelines recommended for reporting animal research, and Sex and Gender in Research

Laboratory animals	C57BL/6J, Myd88fl/fl, Myd88lsl/lsl, Ranklfl/fl, Il1r1fl/fl, Rag1−/−, Myd88−/−, Tlr2−/−, and Tlr4lps-de/lps-del, Dmp1-Cre, Osteocalcin-Cre mice were obtained from the Jackson Laboratory (Bar Harbor, ME, USA). Tlr2fl/fl mice were provided by Drs. Rojas, Harding, and Boom (doi: https://doi.org/10.1101/2021.05.19.444905). All mice were bred and housed under specific-pathogen-free (SPF) conditions except Myd88−/− and Myd88lsl/lsl mice that were bred and housed under SPF conditions with autoclaved feed and acidified water (pH 2.3 to 2.8). All mice were maintained at the Laboratory Animal Resource Center (LARC) of the Indiana University School of Medicine of the Indiana University. The sex and age of mice used (Male and Female, ten to eleven-week-old) are described in manuscript, figures or figure legends. All animals were maintained at a constant ambient temperature of 22-26 degree Celsius, 40-65% of humidity under a 12 h light/dark cycle with free access to food and drink.
Wild animals	The study did not involve wild animals.
Reporting on sex	Sex is mentioned in each graph and legends.
Field-collected samples	The study did not involve samples collected from the field.
Ethics oversight	All mutant mouse lines and experimental procedures were approved by the Institutional Animal Care and Use Committee (IACUC) of the Indiana University School of Medicine and Institutional Biosafety Committee (IBC) of the Indiana University.

Note that full information on the approval of the study protocol must also be provided in the manuscript.
